# N-glycoproteomic profiling reveals aberrant ITGB1 glycosylation in hyperoxia-induced bronchopulmonary dysplasia

**DOI:** 10.1371/journal.pone.0362192

**Published:** 2026-11-10

**Authors:** Cong Zhao, Jie Liu, Chao Tong, Chunyan Chen, Yalan Liu, Yan Chen, Lin Wang, Hua Peng

**Affiliations:** Department of Pediatrics, Union Hospital, Tongji Medical College, Huazhong University of Science and Technology, Wuhan, Hubei, China; University Medical Centre Ljubljana (UMCL) / Faculty of Medicine, University Ljubljana (FM, UL), SLOVENIA

## Abstract

**Background:**

Bronchopulmonary dysplasia (BPD) is a prevalent chronic lung disease in preterm infants, characterized by arrested alveolarization. Extracellular matrix (ECM) remodeling and receptor signaling are critical for alveolarization and are strictly regulated by protein N-glycosylation. However, the specific role of the vital post-translational modification in BPD pathogenesis remains unexplored. The present research aims to delineate a N-glycoproteomic landscape and elucidate the glycosylation-associated pathogenic mechanisms in a murine BPD model.

**Methods:**

To establish BPD models, newborn mice were exposed to continuous hyperoxia (85% O_2_) for 14 days. Lung developmental impairment was quantified using Radial Alveolar Count (RAC) and Mean Linear Intercept (MLI). A comprehensive dual-omics profiling strategy was conducted, utilizing an Orbitrap Exploris 480 platform for global proteomics and a timsTOF Pro mass spectrometer for N-glycoproteomics. Key molecular targets were further validated by lectin histochemistry, Western blot, and AlphaFold-based spatial structure modeling.

**Results:**

Hyperoxia exposure successfully induced BPD-like lung injury, substantiated by a marked decline in RAC coupled with a concurrent elevation in MLI (P < 0.001). Conjoint omics analysis identified 612 N-glycoproteins and 1105 modification sites. After normalizing to global protein abundance, we revealed distinct glycosylation alterations, characterized by the identification of 16 upregulated and 100 downregulated modification sites. Furthermore, lectin histochemistry confirmed an overall reduction and redistribution of core-mannose N-glycans on the cell surface in BPD lungs. Bioinformatics analysis demonstrated that differentially glycosylated proteins were predominantly enriched in ECM-receptor interactions, integrin-mediated cell adhesion, and PI3K-Akt signaling. Subsequent Protein-Protein Interaction network analysis revealed Integrin beta-1 (ITGB1) as a crucial hub node; notably, we identified a marked downregulation of its site-specific N-glycosylation. Spatial modeling pinpointed a crucial, disease-associated aberrant glycosylation site at Asn212, located merely 17.3Å from the MIDAS binding pocket. This site-specific hypo-glycosylation is postulated to unmask the ITGB1 interface, potentially perturbing physiological ligand binding.

**Conclusion:**

This study provides the first exhaustive N-glycoproteomic map of hyperoxia-induced BPD. Our results highlight dysregulated N-glycosylation, particularly the site-specific hypo-glycosylation of ITGB1 at Asn212. Based on computational predictions, it is hypothesized to severely disrupts the ECM-receptor networks and integrin affinity, which correlates with potentially driving alveolarization arrest. These insights provide a novel post-translational perspective on BPD pathogenesis and offer new avenues for future investigation into potential therapeutic targets.

## 1. Introduction

Bronchopulmonary dysplasia (BPD), a prevalent chronic pulmonary pathology in preterm infants, is characterized by arrested alveolarization and compromised microvasculature formation [1,2]. Additionally, it is accompanied with pulmonary inflammation and microvasculature fibrosis [3,4]. BPD arises from multiple factors, including chorioamnionitis, atelectasis, infection, mechanical ventilation, parenteral nutrition, hyperoxia exposure, and free radical damage [4–6]. Apparently, the prophylaxis and therapeutic management of BPD remain challenging due to its complex and elusive pathophysiological mechanisms [7,8]. Further investigation into the pathological characteristics and biological processes of BPD will facilitate a more comprehensive elucidation of the disease, thereby discovering potential new therapeutic targets. In recent years, the continuous development of advanced technologies such as transcriptomics [9,10], proteomics [11–13], metabolomics [14,15], and single-cell RNA sequencing [16] have contributed to elucidating the pathogenesis of BPD. However, classical omics approaches primarily focus on protein abundance, largely overlooking the crucial regulatory roles of post-translational modifications (PTMs) in protein activity, stability, and spatial distribution during dynamic lung development.

Proteins are essential components of organisms, and PTMs of proteins constitute a significant area of protein research [17,18]. Among these, glycosylation of protein, the enzymatic process of attaching sugar chains to proteins [19], is a widespread PTM, affecting over 50% of cellular proteins. Glycosylation encompasses several distinctPTMs, specifically N-linked (N-glycosylation), O-linked (O-glycosylation), C-linked (C-glycosylation), glypiation and phosphoglycosylation [20]. Notably, N-glycosylation at asparagine residues is prevalent in eukaryotes and is the most extensively studied glycosylation form. Studies showed that N-glycosylated proteins are profoundly involved in regulating extracellular matrix (ECM) remodeling, receptor-kinase signaling, and cellular adhesion [21,22]. For instance, the functionality of transmembrane receptors, such as the integrin family, heavily relies on specific N-glycan branches to maintain proper folding and mediate cell-ECM interactions.

Crucially, the hallmark pathologies of BPD are characterized by arrested alveolarization. The structural integrity of the lung scaffold and the spatiotemporal migration of alveolar epithelial cells are strictly governed by ECM components and their cell-surface receptors [23,24]. Under hyperoxic stress, the disruption of the cellular glycosylation machinery may lead to abnormal N-glycan occupancy on these key structural proteins, impairing microvascular formation and alveolar septation. Therefore, investigating the aberrant N-glycosylation landscape is all imperative for unmasking the hidden regulatory mechanisms of BPD. At present, there is a dearth of research examining glycosylated proteins in BPD, with most studies on pulmonary glycosylation focusing on lung cancer [12,25]. Until now, no studies have investigated whether arrested alveolarization is associated with altered N-glycosylated proteins in BPD.

In this study, we carried out a comprehensive quantitative N-glycosylated protein analysis on lung tissue to study glycosylation site changes in mouse models of BPD. Our research findings contribute to an expanded understanding of N-glycosylated proteins in the BPD model, as well as the functions of N-glycosylated proteins during the development of BPD.

## 2. Materials and methods

### 2.1. Experimental animals

Experimental newborn pups in this study were prepared based on the classic model of BPD-like injury [3,26,27]. Adult C57BL/6 mice (7 weeks) for breeding were purchased from Shulaibao Biotechnology Co., Ltd. (Wuhan, China). All mice were raised in specific pathogen-free (SPF) animal rooms at Laboratory Animal Center, Huazhong University of Science and Technology. The environmental temperature was maintained at 25–26°C, and a 12-hour light-dark cycle was implemented. Food and drinking water were available *ad libitum*. Eight-week-old C57BL/6 mice were mated at a male-to-female ratio of 1:3 for natural conception. Newborn mouse pups from dams were randomly divided into two groups, with 5 pups per group. Given that no significant sex differences in body weight and baseline lung morphology were observed on postnatal day 1 (PN1), we have clarified that male and female offspring were pooled for subsequent analyses, with an equal sex distribution across the experimental groups. The BPD model group and the control group were exposed to 85% and 21% oxygen in controlled oxygen chambers, respectively. Anhydrous calcium chloride and quicklime were used to absorb moisture and carbon dioxide. Throughout the 14-day experimental period, the health status and behavior of the mice were monitored daily to minimize distress and alleviate suffering. At the end of the experiment (PN14), all mice were deeply anesthetized with an overdose of isoflurane. Once the complete loss of the pedal withdrawal reflex confirmed a deep surgical plane of anesthesia, euthanasia was achieved via rapid exsanguination through thoracotomy. Subsequently, lung tissues were harvested after lavage with PBS. The excised lungs were then partitioned into two distinct aliquots: one segment was immersed in 4% paraformaldehyde for fixation, whereas the remaining portion was immediately snap-frozen in liquid nitrogen and preserved at −80°C to facilitate downstream analytical procedures (Fig 1A). All experimental protocols were executed in strict adherence to the ARRIVE guidelines, alongside institutional mandates governing the ethical care and utilization of laboratory animals. All animal care and experimental protocols were rigorously reviewed and approved by the Institutional Animal Care and Use Committee of Huazhong University of Science and Technology (IACUC Number: 5160).

**Fig 1:**
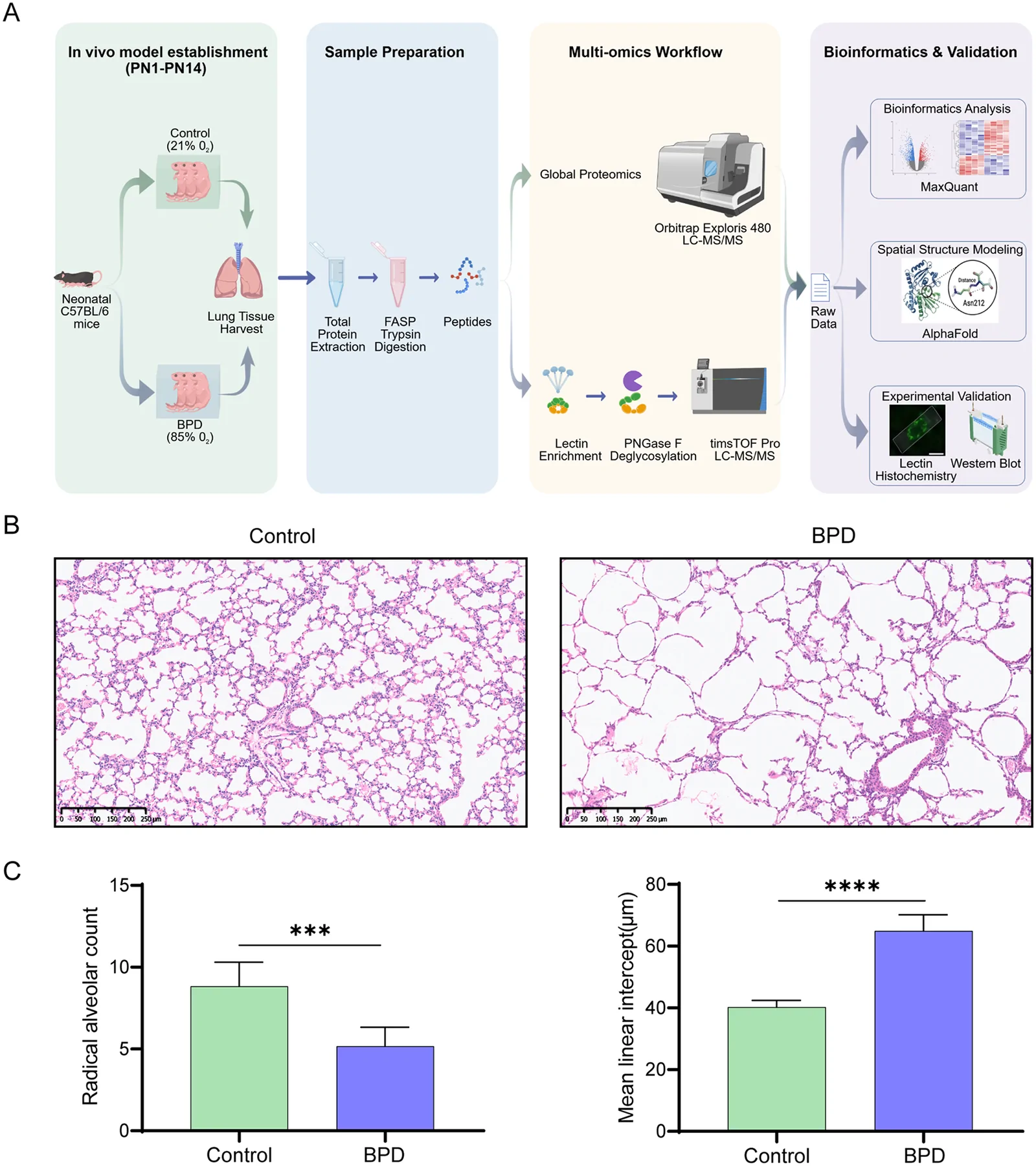
Establishment of animal model. (A) Mouse pups undergo exposure to normal oxygen (21% O_2_) or elevated oxygen levels (85% O_2_) from birth (PN1) until postnatal day 14 (PN14). Following the exposure period, lungs are harvested for glycosylated peptide enrichment. (B) Lung histology of BPD and control groups (Scale bars, 250 μm). The control group shows well-developed alveoli with a trend towards uniform alveolar morphology. The hyperoxia-exposed mice demonstrate impaired alveolar development and simplified alveolar structure. (C) The RAC and MLI are determined to assess lung development. Statistical significance was determined using Student’s t-test. *** P < 0.001 vs. Control, **** P < 0.0001 vs. Control. Created with BioGDP.com.

### 2.2. Histological and morphological analysis

The harvested lung tissues were soaked in 4% paraformaldehyde (Biosharp, China) overnight, and then embedded in paraffin. Using a microtome, the paraffin-embedded tissue was then sliced into thin sections of 4–6 micrometers in thickness. The tissue sections were stained with hematoxylin and eosin (Solarbio, China) for several minutes, dehydrated with alcohol, and rendered transparent with xylene. Finally, the sections were sealed with neutral resin and covered with coverslips for microscopic observation (OLYMPUS BX53, Japan). The radial alveolar count (RAC) was determined by drawing a line from the center of the terminal bronchioles to the nearest connective tissue septum and expressed as the number of alveoli per unit length of the line [28]. Mean linear intercept (MLI) was measured by determining the average distance between the intersections of alveolar septal tissue [29].

### 2.3. Protein extraction, digestion, and N-glycopeptide enrichment

Murine lung tissues were retrieved from liquid nitrogen and lysed followed by sonication. The lysates were centrifuged to obtain the supernatant, and protein concentrations were determined using a bicinchoninic acid (BCA) assay. Protein integrity was assessed by sodium dodecyl sulfate–polyacrylamide gel electrophoresis (SDS–PAGE). Samples that met the quality criteria were subjected to enzymatic digestion using the filter-aided sample preparation (FASP) method [30]. The resulting peptide fractions were collected after centrifugation and used for subsequent proteomic and glycoproteomic analyses.

To obtain information on N-glycosylation sites, the collected peptides were subjected to lectin affinity enrichment. Briefly, peptide samples were incubated with a lectin mixture and subsequently transferred to an ultrafiltration device, where unbound components were removed by centrifugation. Glycopeptides retained on the filter membrane were washed several times with binding buffer, followed by two washes with 40 mM NH_4_HCO_3_ prepared in H_2_^18^O to reduce nonspecific binding. PNGase F was then added to the system, and the reaction was carried out at 37°C for 3h to achieve enzymatic deglycosylation. The deglycosylated peptides were collected by centrifugation, followed by an additional wash to maximize peptide recovery. The resulting peptides were subsequently desalted and concentrated using StageTips and reconstituted in 0.1% formic acid. Crucially, under H_2_^18^O conditions, the hydrolysis of N-linked glycans incorporates an ^18^O isotopic tag into the resulting deamidated asparagine, thereby permitting precise localization of N-glycosylation sites during downstream mass spectrometric interrogation.

### 2.4. LC-MS/MS data acquisition

For N-glycoproteomics, deglycosylated peptides underwent separation via a NanoElute nanoscale liquid chromatography platform (Bruker, Bremen, Germany) coupled to a timsTOF Pro mass spectrometer (Bruker, Bremen, Germany), with data acquisition executed in PASEF mode. Conversely, for global proteomics, peptide fractions were resolved using an EasynLC nanoflow system (Thermo Fisher Scientific) and analyzed on an Orbitrap Exploris 480 instrument, where spectra were recorded in positive-ion data-dependent acquisition (DDA) mode.

### 2.5. Lectin histochemistry

The 4–6μm thick paraffin sections underwent deparaffinization in 100% xylene for a duration of 20 minutes twice and were then hydrated through incubation in graded ethanol solutions (100%, 100%, 95%, 80% and 60%), with each step lasting 5 minutes. Following heat-mediated antigen retrieval, tissue sections were immersed in a 0.3% hydrogen peroxide solution diluted in Tris-buffered saline (TBS; Coolaber, China) and maintained at ambient temperature to suppress endogenous peroxidase activity. Subsequently, non-specific binding sites were blocked by incubating the sections with 5% bovine serum albumin (BSA; Biofroxx, Germany). The sections were incubated with Concanavalin A (ConA), a specific lectin that binds to the core mannose residues of N-glycans, using an, Alexa Fluor™ 488 conjugate (1:50; Invitrogen, Camarillo, CA) overnight at 4°C. The sections were stained using Mounting Medium with antifading agent (containing DAPI) (Solarbio, China). Fluorescence microscopy was conducted utilizing an inverted microscope system (OLYMPUS IX73, Japan).

### 2.6. Western blot

Total proteins were prepared from lung specimens using PBS and PNGase F (Sigma-Aldrich, P7367) was then added to the precipitated product. The resulting protein fractions were subsequently separated via SDS-PAGE and transferred onto 0.45μm nitrocellulose membranes (Cytiva, 10600002, Massachusetts). Following transfer, membranes underwent overnight incubation at 4°C with specific primary antibodies: rabbit polyclonal anti-ITGB1 (1:5000; catalog no. 12594–1-AP, Proteintech) and mouse monoclonal anti-actin (1:20000; catalog no. 66009–1-Ig, Proteintech). After primary antibody incubation, membranes were washed using Tris-buffered saline containing 0.1% Tween-20 (TBST). Detection was achieved by incubating membranes for one hour at room temperature with DyLight 800-conjugated goat anti-rabbit IgG secondary antibodies (1:10000; A23910, Abbkine). Immunoreactive signals were visualized employing an Odyssey CLx infrared imaging system (LI-COR Biosciences, Nebraska). Quantification of the western blots was performed by ImageJ.

### 2.7. Data analysis

The raw mass spectrometry datasets described above were processed for identification and quantification using MaxQuant software (version 1.6.17.0). The database used in this study is uniprot_Mus_musculus_10090_swiss_prot. Digestion parameters were configured for trypsin specificity, with Carbamidomethyl (C) specified as a fixed modification and Oxidation (M) designated as a variable modification. For glycosylomics data, Deamidation18O(N) was additionally included to identify N-glycosylation sites after deglycosylation by PNGaseF. False discovery rates (FDR) were strictly maintained below 1% at both the peptide and protein levels. Quantification relied on Label-Free Quantification (LFQ) intensity values. The screening criteria for differential proteins are set as: |Fold change| > 1.5 and p < 0.05 (Student’s-test), and the screening criteria for differential N-glycopeptides are set as: |Fold change| > 1.2 and p < 0.05 (Student’s t-test). N-glycopeptides, glycoproteins or total proteins that meet the above conditions are defined as differential molecules. All statistical computations were executed utilizing GraphPad Prism 9.5 Software (San Diego, USA). Western blot quantification relied on the unpaired Student’s t-test, with results reported as the means ± SEM. Thresholds for statistical significance were defined as *P < 0.05, ***P < 0.001 and ****P < 0.0001.

### 2.8. Bioinformatics analysis

To comprehensively characterize the biological functions and roles of proteins with differential N-glycosylation modifications as well as total proteins, enrichment analyses were performed on the identified differential proteins and N-glycosylation sites. Functional annotation was primarily conducted using the Gene Ontology (GO) and Kyoto Encyclopedia of Genes and Genomes (KEGG) databases, with p < 0.05 considered statistically significant. The STRING database was used to analyze functional enrichment and protein-protein interactions (PPI) among the differential proteins. The interaction networks were further visualized using Cytoscape, enabling graphical representation of the networks and identification of key nodes. For subcellular localization predictions, we relied on the widely recognized WoLF PSORT. To visualize the spatial localization of identified N-glycosylation sites, the three-dimensional structure of *Mus musculus* Integrin beta-1 (ITGB1, UniProt ID: P09055) was retrieved from the AlphaFold Protein Structure Database. Structural visualization and distance measurements were performed using PyMOL (v2.5, Schrödinger, LLC).

## 3. Results

### 3.1. Hyperoxia impaired pulmonary maturation in neonatal murine models

The HE staining of lung tissue in the control group showed good alveolar structure and consistent alveolar morphology. In contrast, lungs from the BPD model exhibited distinct pathological changes characterized by thinning of the alveolar septa, a sharp decrease in alveolar counts, and marked alveolar enlargement (Fig 1B). Quantitative assessment further indicated that, relative to controls, the BPD group manifested significantly diminished radial alveolar count (RAC) alongside markedly elevated mean linear intercept (MLI) values (Fig 1C). Collectively, these findings substantiate that hyperoxic exposure critically disrupts pulmonary development within the BPD experimental model.

### 3.2. Protein identification and analysis

Compared to the control group, PCA analysis and clustering analysis determined good parallelism and repeatability of the BPD model, with significant heterogeneity observed between the two groups (S1 File). To visualize the statistically significant disparities between the two groups, a volcano plot was generated (S1 File). Relative to the control, the BPD model group exhibited a marked elevation in the abundance of 72 proteins and a significant reduction in 88 other protein species. According to the subcellular localization analysis of these different proteins, the majority of the differentially abundant proteins were localized to cytosol (22.0%), mitochondria (18.2%) and plasma membrane (17.6%) (S1 File). Statistical analysis of the differential proteins was conducted at the second level of GO functional annotation hierarchy. The GO analysis revealed that significantly dysregulated proteins were primarily associated with mitochondrial respiratory chain complex I, lung lobe morphogenesis, NADH dehydrogenase activity. (S1 File). KEGG results indicated that the differential proteins are primarily involved in oxidative phosphorylation, chemical carcinogenesis−reactive oxygen species, ECM receptor interaction and metabolism of xenobiotics by cytochrome P450 (S1 File). The enrichment of KEGG pathway in the top 20 was most abundant in Retrograde endocannabinoid signaling (S1 File). Specific information about the pathway is shown in S1 File.

### 3.3. Identification of N-glycosylation sites and N-glycosylated proteins

Since PTMs, particularly N-glycosylation, strictly dictate protein conformation and function during lung development, we subsequently performed a comprehensive N-glycoproteomic profiling. In order to identify the intra-group and inter-group similarity between the two groups, a clustering analysis of the relative abundance of glycosylation sites was conducted. The results showed pronounced homogeneity within each cohort contrasted with marked heterogeneity between the two groups (Fig 2C). In lung tissue specimens derived from both the BPD model and control groups, the proteomic analysis successfully characterized 612 distinct N-glycosylated proteins encompassing 1,105 specific modification sites. The accompanying volcano plot delineates the comparative distribution of these N-glycosylation loci between the two experimental groups; within this visualization, data points colored red and green denote statistically significant upregulation and downregulation of glycosylation events, respectively. (Fig 2A). Relative to the control group, the BPD model exhibited a marked elevation in the abundance of 20 N-glycosylation sites, concomitant with a substantial reduction across 103 distinct sites. (S1 File). Among them, Low-density lipoprotein receptor-related protein 1 (LRP1, Q91ZX7) emerged as the most prominent candidate, characterized by the presence of 18 glycosylation sites. Integrin alpha 1 (ITGA1, Q3V3R4) was identified with 11 glycosylation sites. According to the subcellular localization analysis of these different proteins, proteins were distributed in the plasma membrane (55.2%), extracellular (24.0%), and endoplasmic reticulum (11.5%) (Fig 2E). The GO analysis indicated that these significantly altered proteins are primarily involved in protein phosphatase binding, apoptotic process and phagocytosis (Fig 2B). KEGG analysis highlighted significant involvement in PPAR signaling pathway, renin-angiotensin system, -and cell adhesion molecules (Fig 2D). Enrichment of KEGG pathway in the top 20 was most abundant in cell adhesion molecules (S1 File). Specific information about the pathway is shown in S1 File.

**Fig 2:**
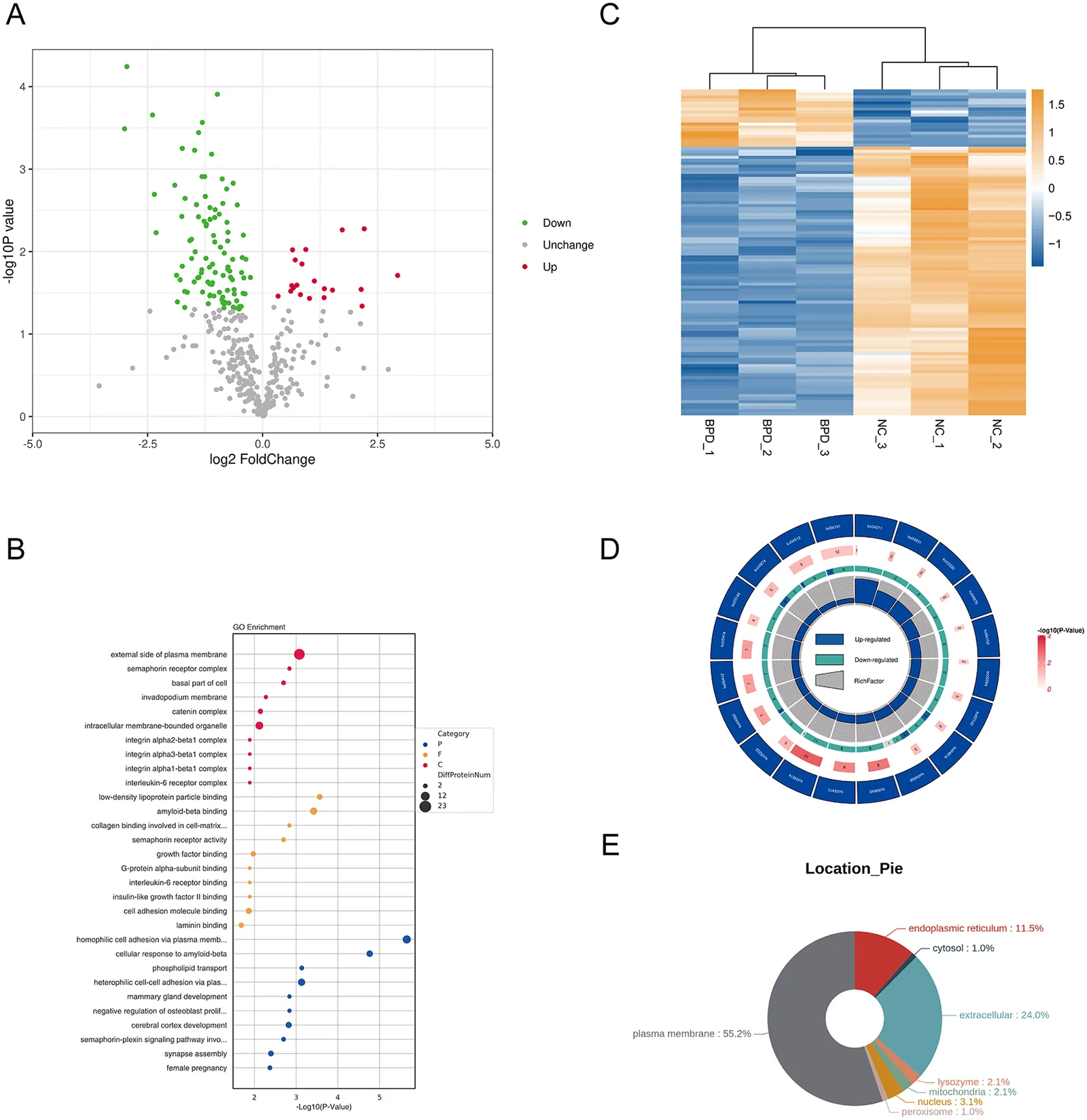
N-glycosylated proteins analysis in the lungs of BPD and control groups. (A) The volcano plot depicts the relative quantity of N-glycosylation sites between BPD and the control group. The red dots representup-regulated proteins, the green dots represent down-regulated proteins, and the gray dots represent no significant difference. (B) GO enrichment analysis of N-glycosylated proteins with significant difference (TOP 10). (C) Clustering heatmap analysis of differential N-glycosylation sites. Each row represents a differential N-glycosylation site. The relative abundance of differential N-glycosylation sitesis indicated by different colors, with yellow representing high expression and blue representing low expression. (D) Enrichment statistical pie chart of KEGG pathways for N-glycosylated proteins with significant difference (TOP 20). (E) Subcellular localization distribution map of differential N-glycosylated proteins.

### 3.4. Decreased N-glycans recognized by ConA in BPD

The distribution of N-glycans was evaluated using ConA, a lectin that specifically binds to core mannose residues of N-glycans. By lectin histochemistry, it was observed that lung tissues from the BPD model exhibited notably decreased N-glycan abundance, as evidenced by substantially weakened ConA fluorescence intensity compared to the control group. Furthermore, N-glycans were distributed on the cell surface, with positive regions also detected within the cytoplasm (Fig 5A).

### 3.5. Conjunctive analysis of proteomics and N-glycoproteomics

To exclude the possibility that the observed alterations in N-glycosylation levels were merely secondary to changes in global protein abundance, we conducted a comprehensive integrative analysis combining proteomic and N-glycoproteomic datasets. By normalizing the glycopeptide intensity to its corresponding total protein expression, the conjunctive analysis of proteomics and N-glycoproteomics was conducted. The volcano plot of differential expression of N-glycosylation sites was constructed (Fig 3A, C). A total of 16 upregulated N-glycosylation sites and 100 downregulated N-glycosylation sites were identified. Among them, 42 glycosylation sites on 39 N-glycoproteins and 62 glycosylation sites on 53 N-glycoproteins were uniquely expressed. GO analysis suggested that the significantly different N-glycosylated proteins identified in the BPD model were mainly involved in cell adhesion mediated by integrin, phospholipid transport, lysosomal transport, and apoptotic process. The molecular function highlighted significant involvement in beta-catenin binding, collagen binding involved in cell-matrix adhesion, and protein phosphatase binding (Fig 3B). Enrichment of KEGG pathway in the top 20 was most abundant in PI3K-Akt signaling pathway and cell adhesion molecules (Fig 3D). Subcellular localization of the proteins revealed that these significant differences in the distribution of N-glycosylated proteins were predominant in plasma membrane (55.1%) and extracellular (24.7%) (Fig 3E).

**Fig 3:**
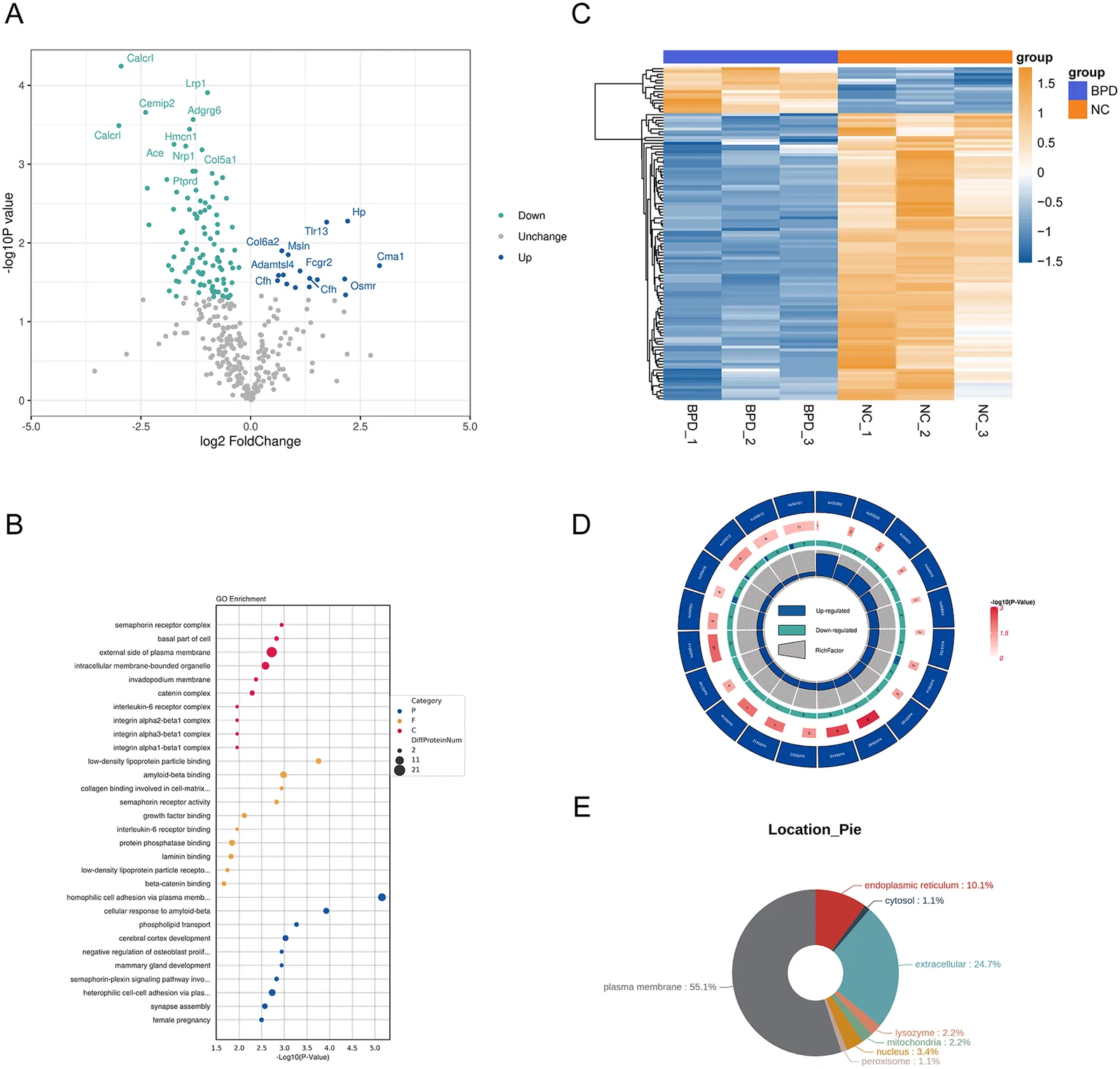
Analysis of N-glycosylation sites after normalization against total protein abundance. (A) The volcano plot depicts the differences in N-glycosylation sites between BPD and the control group. The blue dots represent up-regulated sites, the cyan dots represent down-regulated sites, and the gray dots represent no significant difference. (B) GO enrichment analysis of N-glycosylation sites with significant difference (TOP 10). (C) Clustering heatmap analysis of N-glycosylation sites with significant difference. (D) Enrichment statistical pie chart of KEGG pathways of N-glycosylation sites with significant difference (TOP 20). (E) Subcellular localization distribution map of N-glycosylation sites with significant difference.

### 3.6. PPI network analysis and spatial structural mapping of the hub glycoprotein ITGB1

To further identify key N-glycoproteins involved in BPD, we integrated the proteomic and N-glycoproteomic datasets. Venn diagram analysis revealed an overlap of six proteins that were differentially expressed in both datasets (Table 1). Among these, periostin (POSTN), collagen type XII alpha 1 (COL12A1), and cadherin 13 (CDH13) exhibited significantly increased expression (Fig 4A; S1 File). The interactions between these proteins and the networks formed by these interactions were investigated (Fig 4B) to elucidate their complex interactions.

**Table 1:** The array data of typical N-glycosylation sites of proteins.

**Protein-ID**	**Gene Name**	**GO-ID**	**GO**	**KEGG-ID**	**KEGG**	**Up/Down**
Q08857	Cd36	GO:0060907	positive regulation of macrophage cytokine production	ko03320	PPAR signaling pathway	Down^a^
Q62469	Itga2	GO:0022407	regulation of cell-cell adhesion	ko04145	Phagosome	Down
				ko04151	PI3K-Akt signaling pathway	
				ko04510	Focal adhesion	
				ko04512	ECM-receptor interaction	
P09470	Ace	GO:0048286	lung alveolus development	ko04614	Renin-angiotensin system	Down
				ko04924	Renin secretion	
				ko05142	Chagas disease	
				ko05171	Coronavirus disease - COVID-19	
				ko05410	Hypertrophic cardiomyopathy	
				ko05415	Diabetic cardiomyopathy	
Q3V3R4	Itga1	GO:0051240	positive regulation of multicellular organismal process	ko04151	PI3K-Akt signaling pathway	Down
				ko04510	Focal adhesion	
				ko04512	ECM-receptor interaction	
				ko04640	Hematopoietic cell lineage	
				ko04810	Regulation of actin cytoskeleton	
				ko05165	Human papillomavirus infection	
				ko05410	Hypertrophic cardiomyopathy	
				ko05412	Arrhythmogenic right ventricular cardiomyopathy	
				ko05414	Dilated cardiomyopathy	
Q8R5M8	Cadm1	GO:0050839	cell adhesion molecule binding	ko04514	Cell adhesion molecules	Down
P16406	Enpep	GO:0016477	cell migration	ko04614	Renin-angiotensin system	Down
Q02788	Col6a2	GO:0031012	extracellular matrix	ko04151	PI3K-Akt signaling pathway	Up^b^
				ko04510	Focal adhesion	
				ko04512	ECM-receptor interaction	
				ko04974	Protein digestion and absorption	
				ko05165	Human papillomavirus infection	
Q9WTR5	Cdh13	GO:0043542	endothelial cell migration	No^c^	No	Up

^a^Down: the expression level significantly down-regulated N-glycosylation site

^b^Up: the expression level significantly increased the N-glycosylation site;

^c^No: Not found.

**Fig 4:**
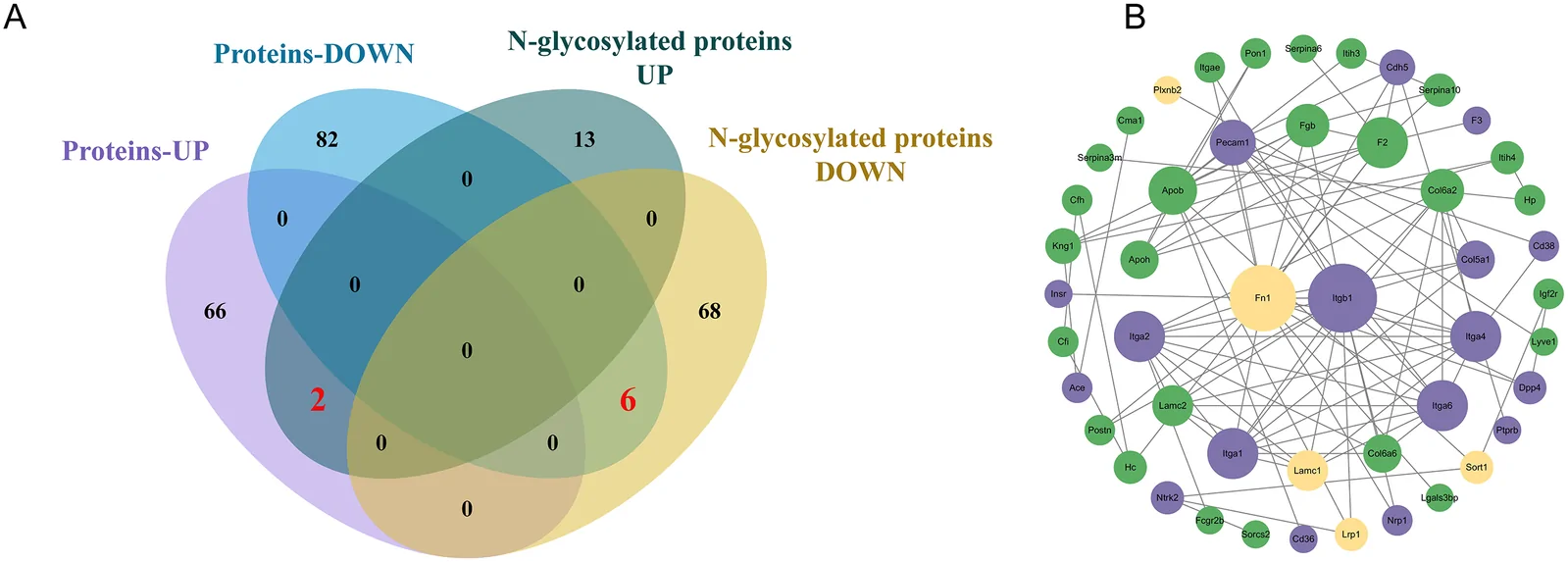
Conjoint analysis of N-glycosylated proteins and proteins with significant difference. (A) Venn diagram of significantly upregulated/downregulated proteins and N-glycosylated proteins. (B) PPI networks of proteins with significantly different sites. In the network, green nodes: upregulated N-glycosylation proteins. Purple nodes: most downregulated N-glycosylation proteins. Yellow nodes: proteins with both up- and downregulated N-glycosylation sites. Node size indicates degree value. (Created by https://cytoscape.org/).

Within PPI framework, ITGB1 emerged as a central hub, exhibiting a disproportionately high connectivity with other network nodes, as evidenced by a degree centrality of 17. Given its indispensable role in ECM-receptor interactions, we focused on ITGB1 for further validation. Before and after PNGase F treatment, it was observed that ITGB1 is N-glycosylated (Fig 5B). Consistently, compared with control group, the level of glycosylation of ITGB1 in BPD model was found to be decreased. The significantly different ITGB1 glycosylation sites identified by glycoproteomics are illustrated in Fig 5C. To explore how aberrant glycosylation might impact ITGB1 function, we performed spatial mapping of the identified sites onto its tertiary structure. We noted that Asn212 resides within a flexible peripheral loop of the I-like domain, a region indispensable for ligand engagement. Intriguingly, structural modeling revealed that the Asn212 side chain is situated a mere 17.3 Å from the MIDAS binding pocket (Fig 5D-E). In contrast, another site, Asn669, was localized to the Calf-2 domain, in proximity to the C-terminal transmembrane stalk. The strategic positioning of Asn212 suggests a potential role in modulating the accessibility of the integrin binding interface. This structural insight implies that the pathological hypoglycosylation at Asn212 during BPD might abnormally expose the MIDAS pocket. We hypothesize that this could potentially dysregulate its interaction with ECM ligands and contributing to arrested alveolarization.

**Fig 5:**
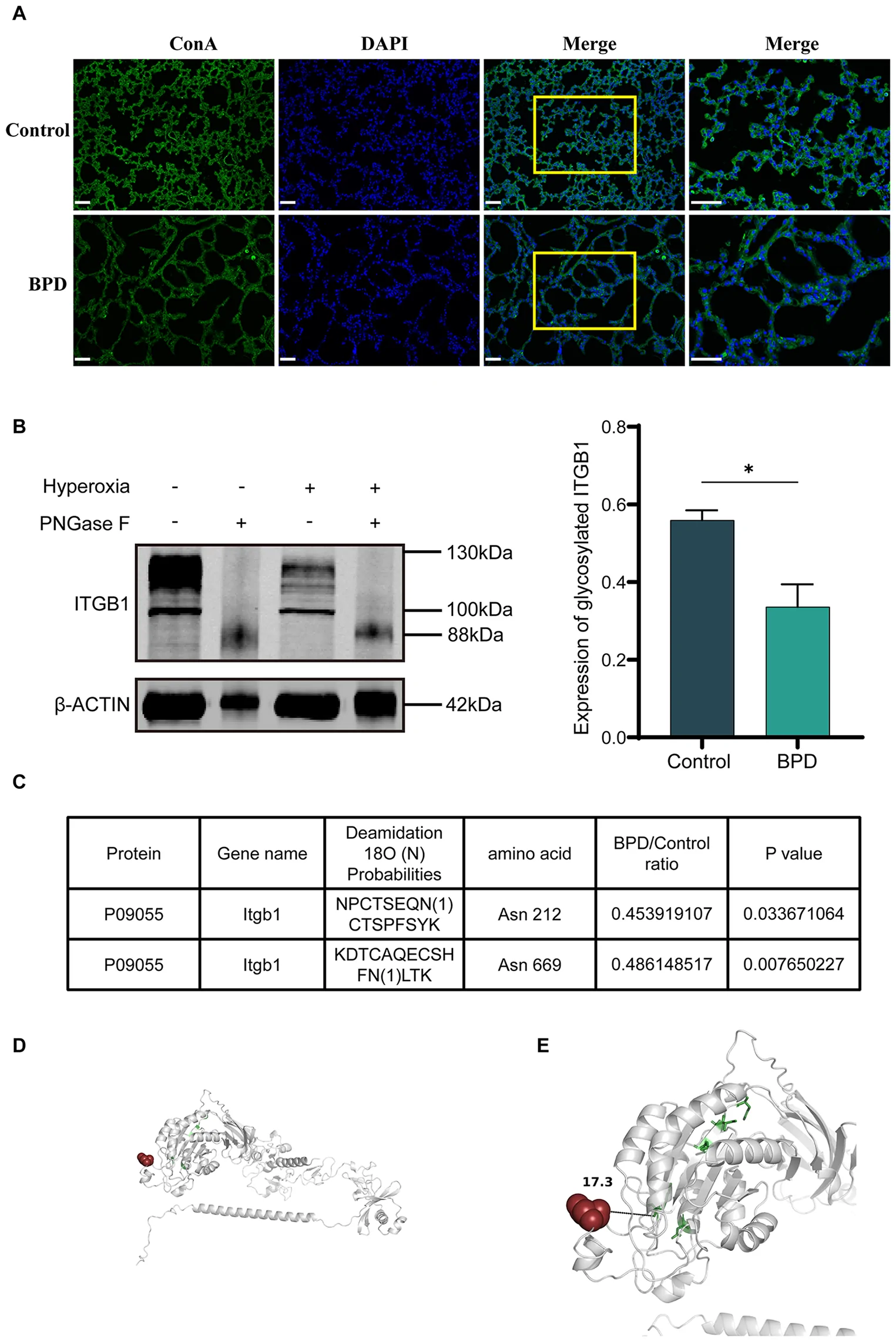
N-glycans was recognized by ConA. (A) Immunofluorescence assay reveales the presence of ConA (shown in green) in lung samples from both the control and BPD groups. The ConA signal is remarkably diminished in the BPD group. The nuclei are stained with DAPI (shown in blue). (Scale bars, 50 μm). (B) Western Blot is used to detect the expression of ITGB1 in lung tissue before and after PNGase F treatment. Statistical significance was determined using Student’s t-test. *P < 0.05. (C) Differential ITGB1 glycosylated sites. (D)Overview of the 3D structure of mouse ITGB1 (gray cartoon). The location of Asn-212 is indicated. (E) Zoomed-in view of the I-like domain ligand-binding pocket. The significantly regulated site Asn-212 (ruby sticks) and the MIDAS motif (green sticks) are highlighted. The spatial distance between the two regions is measured as 17.3Å (dashed line). Note: Structural visualization was performed using PyMOL 3.1.

## 4. Discussion

Advances in neonatal intensive care, particularly the optimization of mechanical ventilation, have significantly improved the survival rate of extremely preterm infants. However, the overall incidence of BPD remains persistently high, accompanied by evolving pathological features characterized predominantly by arrested alveolarization [31]. The primary pathological characteristics of BPD include a reduction in alveoli number, coupled with simplified alveolar structure and aberrant development of the pulmonary microvasculature [4,32,33]. In this study, we observed that the mice exhibited thinning of alveolar septa and simplified structure, indicating impaired alveolarization after hyperoxia exposure for 14 days [1,32]. Impaired lung development confirmed BPD-like pathological changes in the mice exposed to hyperoxia.

Next, we explored and compared the proteomic and N-glycoproteomic datasets between the control group exposed to 21% O_2_ and the BPD group exposed to 85% O_2_ the two groups. Our findings indicate a statistically significant elevation in the abundance of 72 proteins within the BPD model relative to the control group. Among the significantly upregulated proteins, several key enzymes including AKR1B8, CKMT1, and EPHX1 were identified. These alterations primarily reflect the severe oxidative stress (OS) and mitochondrial dysfunction classically triggered by hyperoxia, which collectively impair cellular viability and pulmonary microvascular development [33–35]. While such metabolic disruptions underscore the acute cellular toxicity of hyperoxia, the long-term alveolarization arrest in BPD is associated with ECM remodeling and aberrant receptor signaling. Therefore, rather than solely focusing on global protein abundance, we shifted our primary focus to the crucial post-translational modifications, specifically N-glycosylation, which directly dictate cell-matrix interactions.

During the development of alveoli, mitochondrial respiratory chain complex I in alveolar epithelial cells is necessary [36]. Extensive prior research has established that exposure to hyperoxic conditions markedly compromises mitochondrial functionality within pulmonary tissues, which is closely associated with the dysfunction of complex I [37]. Abnormal loss of mitochondrial respiratory chain complex I can lead to injury of the alveolar wall and structural abnormalities [36,38]. In mammals, mitochondrial respiratory chain complex I is responsible for regenerating mitochondrial NAD^+^ to maintain tricarboxylic acid (TCA) cycle. At the same time, it produces superoxide to regulate the development of alveoli after birth [36]. In this study, GO analysis suggests that mitochondrial respiratory chain complex I may have an important relevant role in the BPD model. In addition, proteomic analysis suggested that the expression levels of multiple proteins such as NDUFA2, NDUFA12, NDUFV1 and NDUFS1 related to the mitochondrial respiratory chain [39] were significantly reduced. This indicates that damage in the mitochondrial respiratory chain may be one of the important mechanisms of BPD, providing a new direction for subsequent research.

While altered protein abundances reflect hyperoxia-induced metabolic and mitochondrial disruptions, the progression of BPD is also associated with ECM abnormalities that reshape pulmonary architecture [24]. Recognizing the pivotal role of post-translational modifications, specifically N-glycosylation, in regulating membrane receptor activity and cell-matrix engagement, we extended our analysis to include N-glycoproteomics. This enabled us to explore how hyperoxic stress-induced modifications alter key proteins involved in alveolar maturation. Combined with the N-glycoproteomic analysis, we found that differential N-glycoproteins are mainly localized in the plasma membrane of cell. Notably, while lectin histochemistry revealed a macroscopic reduction in overall high-mannose/core-mannose type N-glycans on the cell surface in the BPD model, our quantitative N-glycoproteomics pinpointed specific microscopic dynamics, identifying both down-regulated (100 sites) and up-regulated (16 sites) modifications. This apparent discrepancy suggests that hyperoxic stress broadly impairs the global cellular N-glycosylation machinery, yet elicits targeted compensatory hyper-glycosylation at specific sites. This highlights the indispensability of MS-based site-specific profiling, which unmasks critical granular regulatory mechanisms that are typically obscured by global staining approaches. Subsequent GO analysis of these differentially N-glycosylated proteins suggested a strong correlation with ECM-receptor interactions. Extracellular matrix formation and remodeling are essential processes regulating alveolar development [24,40,41]. Matrix remodeling is necessary for alveolar development or otherwise leads to alveolar dysplasia [42]. It has also been confirmed in blood samples from children with BPD [43]. Based on the conjunctive analysis, we speculate that hyperoxia-induced oxidative stress not only dampens the overall expression of integrins but also fundamentally alters their functional mechanics via aberrant N-glycosylation. These improperly glycosylated receptors, particularly ITGB1, may subsequently misguide ECM-receptor interactions, which correlates with a shift in the pulmonary microenvironment from normal alveolarization to pathological fibrosis and apoptosis.

Multiple integrin family proteins were significantly differentially expressed in the BPD model. In the analysis of differential N-glycosylated proteins, ITGA1 is identified as a differentially N-glycosylated protein. Specifically, a total of 11 N-glycosylation sites were identified in ITGA1, among which 7 sites exhibited statistically significant alterations. Integrins are important transmembrane proteins that regulate cell adhesion, migration and proliferation by interacting with ECM [44]. PPI analysis suggests that ITGB1 has more connections to other network nodes and plays an important role in differentially expressed N-glycosylated proteins. Studies have shown that ITGB1 is necessary for alveolar formation, loss of which can lead to bronchial branch defects and disorders of epithelial cell differentiation and maturation in mice [44]. Prior investigations have established that αvβ6 integrin drives aberrant pulmonary morphogenesis and fibrotic progression in neonatal murine models [45]. Conversely, genetic ablation of the β6 subunit significantly enhances alveolar development in mice. Fetal defects in mice can lead to spontaneous inflammation, but during the neonatal period, it can reduce pulmonary inflammation, improving alveolar development and protecting neonatal mice from damage of BPD-like disease [46]. Structurally, the 17.3Å spatial gap between Asn212 and the MIDAS motif places the glycosite in a prime position to regulate ITGB1 activity. While Asn212 resides outside the immediate metal coordination sphere, its proximity falls well within the 20–30Å radius typical of complex N-glycans, allowing for effective steric shielding of the binding pocket. We postulate that the pathological hypo-glycosylation of Asn212 might exposes the ITGB1 interface by reducing steric hindrance, potentially enhancing its binding affinity for ligands such as fibronectin to promote leukocyte infiltration and fibroblastic activation. We hypothesize that this hyperactivation and abnormal cell-matrix interaction could be associated with pathological leukocyte recruitment and excessive fibroblastic activation, though whether this is a primary cause or a secondary consequence of hyperoxic injury remains to be elucidated. The continuous and abnormal engagement of integrins with the ECM can trigger a fibrotic cascade and aberrant matrix deposition, which rigidly disrupts the delicate microenvironment required for secondary septation, ultimately hindering normal alveolarization in BPD [23]. This site-specific loss of glycan occupancy likely functions as a molecular rheostat, fine-tuning integrin affinity via allosteric modulation of the I-like domain periphery. In this study, quantitative Western blot analysis demonstrated that the glycosylation level of ITGB1 were significantly decreased in the BPD group compared with the control group. Therefore, whether the role of integrin family proteins in BPD is caused by glycosylation deserves further study.

Despite the novel findings, we acknowledge several limitations in the present study. First, while the hyperoxia-induced murine model classically mimics the alveolar arrest seen in BPD, it cannot fully capture the multifactorial pathogenesis of human preterm infants. The lack of validation in human clinical samples, such as bronchoalveolar lavage fluid (BALF), limits the immediate clinical translation of our N-glycoproteomic signatures. Second, although we proposed a compelling structural hypothesis regarding the hypo-glycosylation of ITGB1 at Asn212 and its potential role in unmasking the ligand-binding interface to trigger fibrotic cascades, this remains a hypothesis-generating prediction based on structural modeling (AlphaFold) and omics data. The lack of direct functional validation constitutes a limitation. Importantly, our current data demonstrate an association between altered N-glycosylation and BPD, but it remains unclear whether these glycosylation abnormalities are a cause of alveolarization arrest or a consequence of hyperoxia-induced lung injury. In vitro and in vivo functional assays are required to experimentally confirm whether Asn212 hypo-glycosylation directly alters ITGB1 activity, ECM interactions, and lung alveolarization in future investigations. Currently, the structural modeling of Asn212 remains hypothesis-generating, and we cannot conclusively assert a causal role for Asn212 hypo-glycosylation in BPD pathogenesis without further functional evidence. Third, our quantitative N-glycoproteomics was performed on whole lung tissue homogenates, which inevitably masks the profound cellular heterogeneity of the developing lung. It remains to be elucidated whether these aberrant ITGB1 glycosylation events predominantly occur in alveolar epithelial cells or in interstitial fibroblasts. Future investigations employing single-cell or spatial glycoproteomic technologies will be crucial to deciphering the precise cell-type-specific molecular dynamics during BPD pathogenesis.

## 5. Conclusions

In summary, this study delineates the first N-glycoproteomic landscape of a murine BPD model, uncovering an aberrant glycosylation network of transmembrane receptors centered around ITGB1. We postulate that hypo-glycosylation at critical residues, such as Asn212, is associated with pathological ECM hyper-interaction and subsequent fibrosis through a steric deshielding effect, which correlates with alveolarization arrest. These findings not only provide a novel post-translational modification perspective on the complex pathogenesis of BPD, but also highlight that further functional studies will clarify whether targeting specific glycosylation pathways or impeding aberrant integrin-ligand engagements could eventually serve as promising therapeutic strategies for preventing and treating neonatal BPD.

## Supporting information

S1 File Supplementary figures and tables. This file contains Figure S1 (Proteomic analysis in the lungs of BPD and control groups), Figure S2 (KEGG pathway enrichment analysis of proteins with significant difference), Figure S3 (KEGG pathway enrichment analysis of N-glycosylated proteins with significant difference), Table S1 (Significant difference modification sites statistics), and Table S2 (The venn diagram analysis of differentially expressed proteins).
(PDF)

S2 File Raw uncropped and unadjusted Western blot and gel images. This file contains all original raw blot/gel images underlying figures presented in the manuscript. Each image is annotated with the corresponding figure panel, molecular weight marker and sample loading order.
(PDF)
