# Supplementary material for: N-glycoproteomic profiling reveals aberrant ITGB1 glycosylation in hyperoxia-induced bronchopulmonary dysplasia
Source: PLoS One. 2026 Nov 10;21(11):e0362192. doi: 10.1371/journal.pone.0362192 (PMC13750597; doi:10.1371/journal.pone.0362192)
Supplement: Supplementary file 1 [file pone.0362192.s001.pdf]

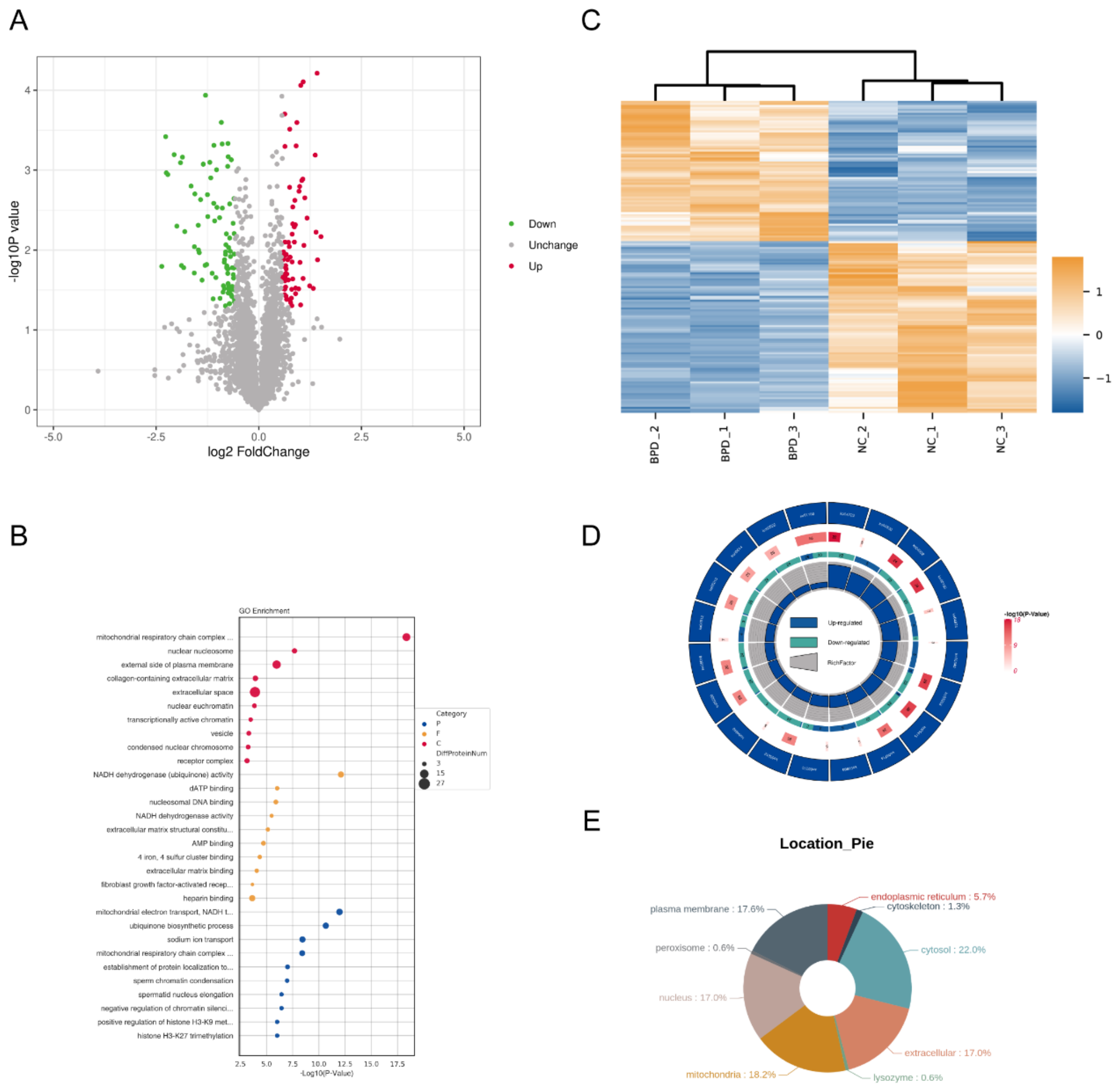

S1 Fig. Proteomic analysis in the lungs of BPD and control groups.

(A) The volcano plot depicts the differences in protein expression between BPD and the control group. The red dots represent up-regulated proteins, the green dots represent down-regulated proteins, and the gray dots represent no significant difference. (B) GO enrichment analysis of proteins with significant difference (TOP 10). (C) Clustering heatmap analysis of proteins with significant difference. (D) Enrichment statistical pie chart of KEGG pathways of proteins with significant difference (TOP 20). (E) Subcellular localization distribution map of differential proteins.

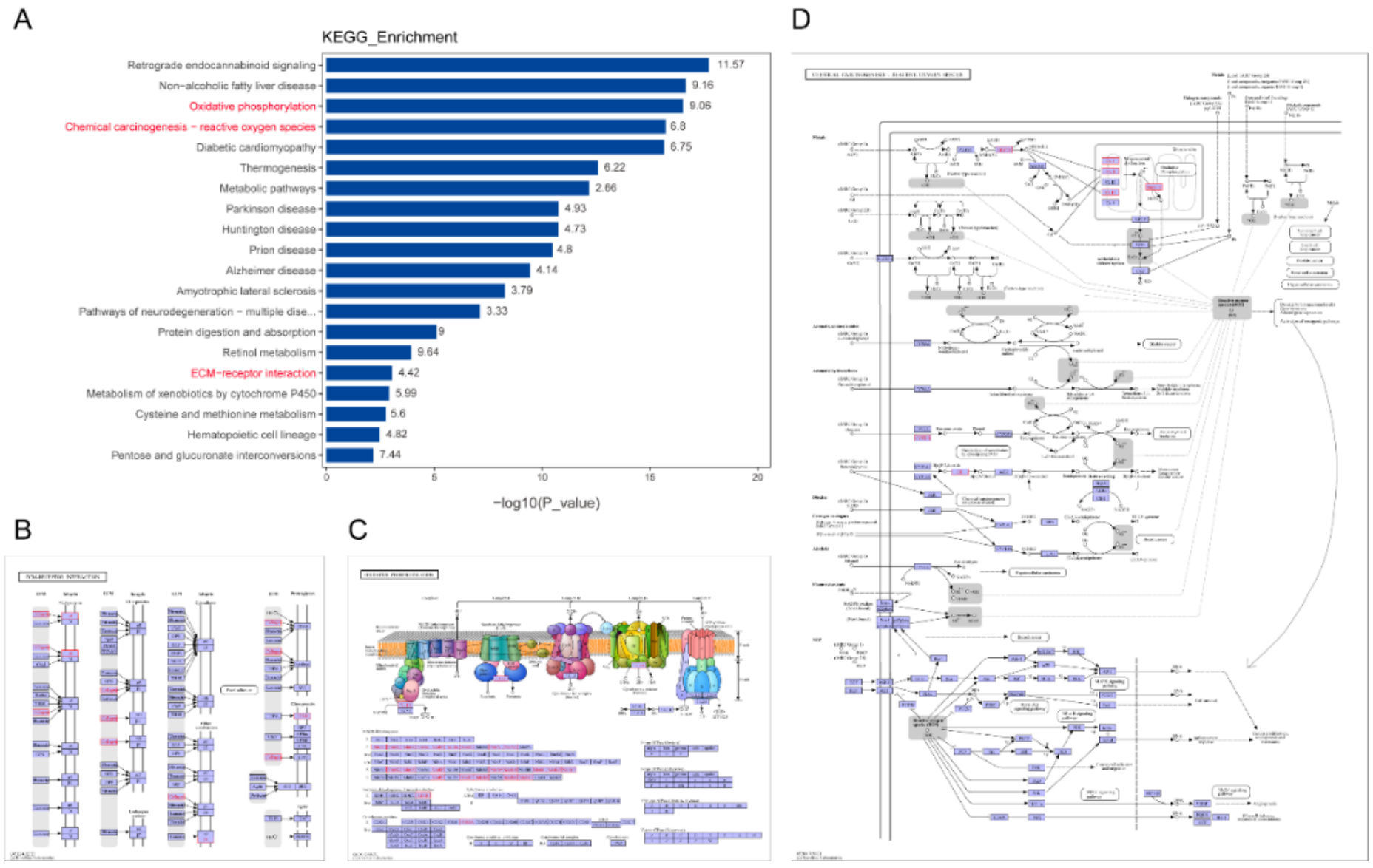

S2 Fig. KEGG pathway enrichment analysis of proteins with significant difference.

(A) Enrichment statistical bar chart of KEGG pathways of proteins with significant difference (TOP 20). (B-D). KEGG pathways: ECM-receptor interaction, oxidative phosphorylation and chemical carcinogenesis-reactive oxygen species.

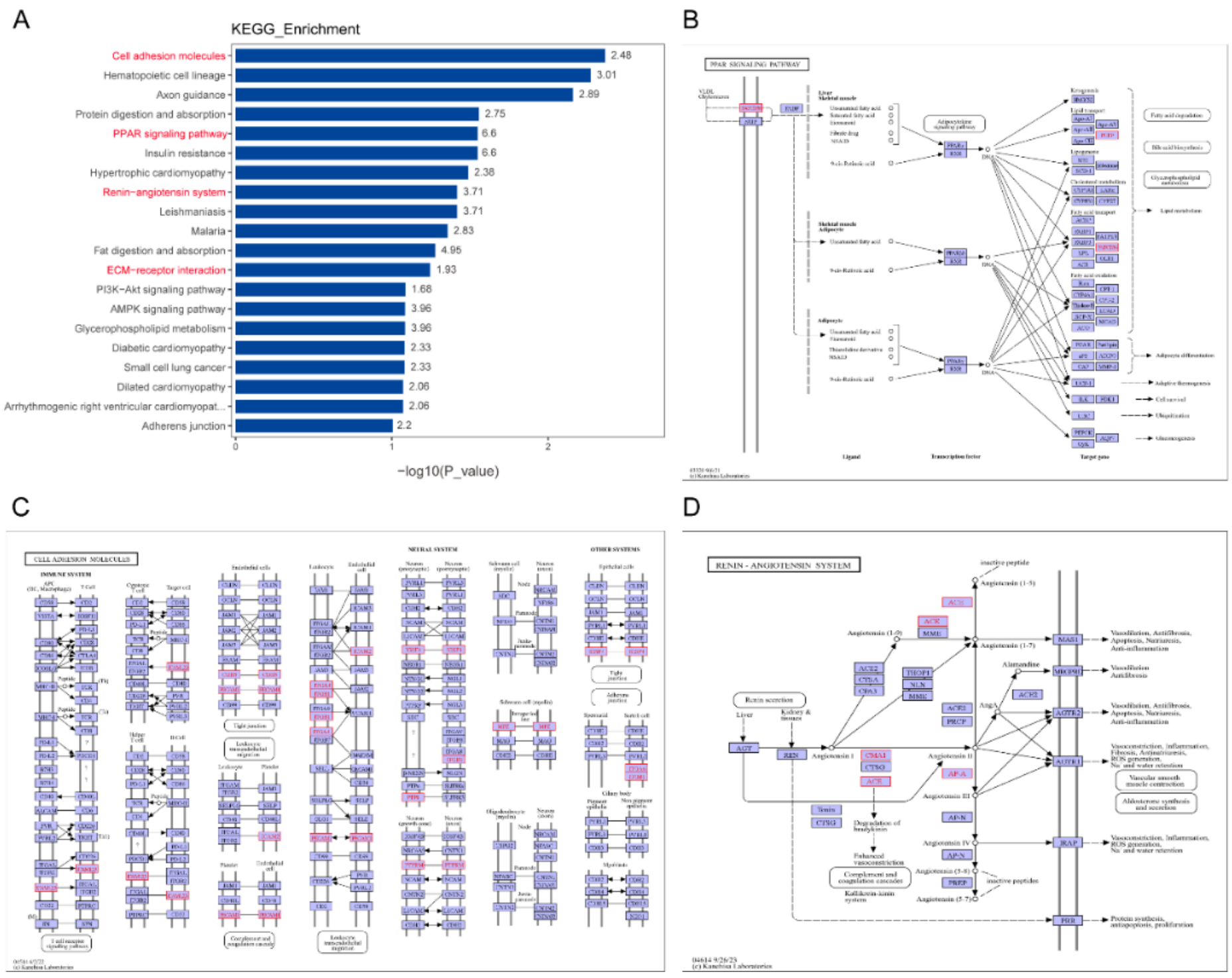

S3 Fig. KEGG pathway enrichment analysis of N-glycosylated proteins with significant difference.

(A) Enrichment statistical bar chart of KEGG pathways of N-glycosylated proteins with significant difference (TOP 20). (B-D). KEGG pathways: PPAR signaling pathway, cell adhesion molecules, renin-angiotensin system.

S1 Table. Significant difference modification sites statistics.

Proteins	Gene Names	Fasta headers	Positions	Localization prob	Score diff	PEP	Score	Number	Amino	Dear	Deamidat	Position	Charge	Mass	id	Protein	Intensity BPD_D14_1	Intensity BPD_D14_2	Intensity BPD_D14_3	Intensity BPD_NC_1	Intensity BPD_NC_2	Intensity BPD_NC_3	Average BPD_D14	Average BPD_NC	BPD_D14 / BPD_NC	Regulation	P valu
P21844	Cma1	sp|P21844|CMA1_MOUSE	80	1	56.3783	0.000129	74.269	1	N	SITVI	SITVLLGA	10	2	1.65	294	223	132940	103240	79737		16190	11323	105306	13756.5	7.654975	UP	0.019
Q61646	Hp	sp|Q61646|HPT_MOUSE	182	1	177.409	3.4E-132	177.41	1	N	VVLI	VVLHPN(	6	3	0.35	647	497	1.7E+07	1.2E+07	1E+07	2797200	2422800	3278500	1.3E+07	2832833	4.627993	UP	0.005
Q60677	Itgae	sp|Q60677|ITAE_MOUSE	1086	1	80.6231	2.44E-07	80.623	1	N	DVSI	DVSELPIL	14	2	2.13	572	459	66742	49226	35002	10828	11644		50323.3	11236	4.478759	UP	0.046
O70458	Osmr	sp|O70458|OSMR_MOUS	74	0.989585	19.7779	2.19E-15	90.376	1	N	LN(0	LN(20)ISN	2	2	1.01	138	94	60256	47563		8738.6	15668		53909.5	12203.3	4.417617	UP	0.029
Q6R5N8	Tlr13	sp|Q6R5N8|TLR13_MOUS	152	1	105.24	6.22E-47	140.57	1	N	GLEN	GLEN(110	4	2	1.39	746	560	51173	53956	57437	10022	22602		54188.7	16312	3.322012	UP	0.005
Q03734	Serpina3m	sp|Q03734|SPA3M_MOU	184	1	69.2108	8.02E-07	108.91	1	N	LIND	LIN(-69)D	8	2	0.9	473	406	895660	1107000		378660	528710	140250	1001330	349207	2.867442	UP	0.029
P06909	Cfh	sp|P06909|CFAH_MOUSE	773	1	48.7007	0	282.16	1	N	LTEF	LTEFTHN(	7	2	-0.5	198	135	1824300	1205900	1075300	483640	708730	425390	1368500	539253	2.537768	UP	0.028
Q9WTR5	Cdh13	sp|Q9WTR5|CAD13_MOU	530	0.999956	43.5691	4.6E-206	135.89	1	N	DPA(	DPAGWLS	12	2	-0.7	1076	824	921640	519720	554780	297530	240580	251610	665380	263240	2.527655	UP	0.036
P08101	Fcgr2	sp|P08101|FCGR2_MOUS	166	1	47.5611	0.000713	105.36	1	N	YHH	YHHYSSN	7	2	-0.5	206	144	233960	171280	206630	79849	107060		203957	93454.5	2.182417	UP	0.023
Q8K007	Sulf1	sp|Q8K007|SULF1_MOUS	197	0.999517	33.1623	1.23E-12	95.138	1	N	DYF	DYFTDLIT	9	2	1.8	854	646	90555	103170			55150	40393	96862.5	47771.5	2.027621	UP	0.037
Q60847	Col12a1	sp|Q60847|COCA1_MOU	2530	1	43.272	7.38E-17	130.31	1	N	MLE	MLEAYN(	6	2	0.32	576	462	1682100	2226100	1737900	1141500	853500	948420	1882033	981140	1.918211	UP	0.009
Q61468	Msln	sp|Q61468|MSLN_MOUS	488	1	139.705	7.2E-118	165.02	1	N	ACS	ACSAFQN	7	2	-1.7	614	484	510460	552540	506630	392520	266210	208620	523210	289117	1.809685	UP	0.014
Q07797	Lgals3bp	sp|Q07797|LG3BP_MOUS	543	1	110.522	1.73E-46	141.44	1	N	APIP	APIPTALD	10	2	-0.9	495	416	1728400	2134000	1368600	995360	1132000	829080	1743667	985480	1.769358	UP	0.033
Q80T21	Adamtsl4	sp|Q80T21|ATL4_MOUSE	454	1	65.1561	0.003554	84.368	1	N	LVSC	LVSGN(65	5	2	0.31	761	574	273260	284800	221170	153760	156190		259743	154975	1.676034	UP	0.026
Q02788	Col6a2	sp|Q02788|CO6A2_MOU	800	1	91.2447	1.18E-21	91.245	1	N	N(1)I	N(91)MTL	1	2	-2.4	470	402	844640		869640	441690	525100	605970	857140	524253	1.634973	UP	0.013
Q03145	Epha2	sp|Q03145|EPHA2_MOUS	436	1	77.3584	2.22E-22	124.46	1	N	TAS\	TASVSIN(	7	2	-2.2	472	404		416460	345360	223960	275320	220320	380910	239867	1.588007	UP	0.028
Q62009	Postn	sp|Q62009|POSTN_MOU	601	1	155.445	1E-125	172.1	1	N	GVN	GVN(160)	3	2	1.58	668	507	5783800	6690800	5280600	4067900	3862100	3381900	5918400	3770633	1.569604	UP	0.01
P06909	Cfh	sp|P06909|CFAH_MOUSE	1225	0.999997	54.9106	1.4E-43	151.22	1	N	CIN(	CIN(55)G	3	2	3.46	196	135	221170	158390	182690	109690	130830	121770	187417	120763	1.551934	UP	0.026
P06684	C5	sp|P06684|CO5_MOUSE	733	1	119.762	2.37E-10	133.76	1	N	AFN	AFN(120)	3	2	0.49	189	130	865530	1009500		703440	602970	532890	937515	613100	1.529139	UP	0.03
Q07113	Igf2r	sp|Q07113|MPRI_MOUSE	1532	1	67.8807	6.97E-18	100.84	1	N	AGIN	AGIN(68)	4	2	0.22	487	415	1568800	1940500	1811100	1473600	1415800	1324100	1773467	1404500	1.262703	UP	0.035
Q3TDQ1	Stt3b	sp|Q3TDQ1|STT3B_MOU	638	1	47.5611	8.57E-10	108.3	1	N	AMS	AMSSN(4	5	2	-0.2	510	423	964560	1161500	1055500	1264000	1290900	1267700	1060520	1274200	0.832303	DOWN	0.021
B2RXS4	Plxnb2	sp|B2RXS4|PLXB2_MOUS	1103	1	99.6681	0	146.75	1	N	TEA(	TEAGVFE	16	2	1.19	33	17	357230	312920	341430	424370	408010	468780	337193	433720	0.777445	DOWN	0.012
Q9R0E2	Plod1	sp|Q9R0E2|PLOD1_MOU	539	1	67.4639	0.010454	106.93	1	N	YIHE	YIHEN(67	5	2	-4.3	1066	815	520350	650540	513900	772850	744040	676830	561597	731240	0.768006	DOWN	0.033
Q62470	Itga3	sp|Q62470|ITA3_MOUSE	925	1	84.2656	5.4E-152	109.16	1;2	N	CVW	CVWLECF	13	2	0.45	682	520	552900	674880	613410	801180	892420	750900	613730	814833	0.753197	DOWN	0.021
Q91ZX7	Lrp1	sp|Q91ZX7|LRP1_MOUSE	730	1	76.8501	0	238.54	1	N	IETIL	IETILLN(7	7	2	-0.5	936	705	2132100	2054900	2229700	2936600	3028700	2608700	2138900	2858000	0.74839	DOWN	0.006
O35887	Calu	sp|O35887|CALU_MOUSE	131	1	200.626	0	215.11	1	N	N(1)/	N(200)AT	1	2	-1.1	117	75	740250	771270	962580	1108300	1196100	1013800	824700	1106067	0.745615	DOWN	0.032
Q810U4	Nrcam	sp|Q810U4|NRCAM_MOU	308	1	78.1783	5.18E-11	130	1	N	EDG	EDGMLP	8	2	0.49	785	584	142830	136120	140800	167510	201150	201310	139917	189990	0.736442	DOWN	0.012
O08532	Cacna2d1	sp|O08532|CA2D1_MOUS	824	1	88.4125	2.6E-58	138.71	1	N	IDVN	IDVN(-88	9	2	-2.1	76	48	283510	338420	285480	460690	396480	388350	302470	415173	0.728539	DOWN	0.018
Q9JKR6	Hyou1	sp|Q9JKR6|HYOU1_MOU	515	1	59.6454	4E-06	97.277	1	N	VFG	VFGSQN(	6	2	-1.2	1046	796	2156400	3001700	2478900	3741500	3751700	3046800	2545667	3513333	0.724573	DOWN	0.046
P06800	Ptprc	sp|P06800|PTPRC_MOUS	429	0.939133	11.8835	5.56E-97	118.26	1	N	SLPN	SLPN(-12	5	2	-2.8	194	133	284810	266320	296050	472980	333970	392880	282393	399943	0.706083	DOWN	0.046
Q2PZL6	Fat4	sp|Q2PZL6|FAT4_MOUSE	2923	1	88.1134	0.000549	94.804	1	N	IN(1)	IN(88)ATT	2	2	-2.5	506	420	115610	141850	161670	185260	232380	179800	139710	199147	0.701543	DOWN	0.05
O08532	Cacna2d1	sp|O08532|CA2D1_MOUS	781	1	115.194	5.9E-132	128.41	1	N	SLDN	SLDN(-13	15	2	-0.2	78	48	167170	196850	206810	291050	316280	221720	190277	276350	0.688535	DOWN	0.049
Q8BXN9	Tmem87a	sp|Q8BXN9|TM87A_MOU	127	1	60.8918	0.000313	105.36	1	N	LFQI	LFQN(61)	4	2	-0.1	823	619	731500	684920	607720	926850	1021700	1016700	674713	988417	0.68262	DOWN	0.003
Q9ER41	Tor1b	sp|Q9ER41|TOR1B_MOU	64	1	87.7273	3.67E-53	121.86	1	N	FTEC	FTECCHE	12	3	0.15	1021	778		157940	165090	234960	269610	211250	161515	238607	0.676909	DOWN	0.04
Q91ZX7	Lrp1	sp|Q91ZX7|LRP1_MOUSE	1576	1	61.1942	1.16E-07	100.84	1	N	LHKI	LHKDN(6	5	2	-1.1	933	705	276740	322150	230260	448180	398370		276383	423275	0.652964	DOWN	0.033
O70423	Aoc3	sp|O70423|AOC3_MOUS	592	0.999988	49.3912	2.4E-117	187.23	1	N	YLYL	YLYLASN(	7	2	-1.5	136	93	379070	375270	312810	440510	603130	595130	355717	546257	0.65119	DOWN	0.029
Q9QZC2	Plxnc1	sp|Q9QZC2|PLXC1_MOU	407	0.999998	57.8279	1.7E-102	125.75	1	N	VVL(	VVLGEN(	6	2	-2.8	1060	809	239770	276670			426160	372860	258220	399510	0.646342	DOWN	0.049
Q8C310	Robo4	sp|Q8C310|ROBO4_MOU	247	1	70.2982	8.78E-57	109.47	1	N	IQLE	IQLEN(70	5	2	-3	837	629	207780	268030	270120	451030	406210	302180	248643	386473	0.643365	DOWN	0.047
Q00560	Il6st	sp|Q00560|IL6RB_MOUSE	225	1	87.7541	5.9E-187	164.79	1	N	VKP	VKPTPPY	8	3	-1.5	458	393	234080	203100	232390	364410	355170	323240	223190	347607	0.642076	DOWN	0.001
Q8CJH3	Plxnb1	sp|Q8CJH3|PLXB1_MOUS	1251	1	78.864	1.35E-16	124.06	1	N	YTSD	YTSDPN(	6	2	-1.5	849	642	120620	138720	117910	215790	209210	167160	125750	197387	0.637074	DOWN	0.012
Q8VI56	Lrp4	sp|Q8VI56|LRP4_MOUSE	1415	0.999743	35.9021	7.56E-80	107.6	1	N	ADLI	ADLN(36)	4	2	-0.2	915	691	73802	84378	74702	106400	145310	115160	77627.3	122290	0.634781	DOWN	0.022
Q9Z1X2	Ptdss2	sp|Q9Z1X2|PTSS2_MOUS	159	1	111.42	8.59E-51	128.41	1	N	DYG	DYGGN(-	13	2	-0.5	1119	853		133960	115040	217560	209460	177880	124500	201633	0.617457	DOWN	0.021
B2RXS4	Plxnb2	sp|B2RXS4|PLXB2_MOUS	451	1	74.62	0	224.97	1	N	QDL	QDLALSG	8	2	1.26	30	17	105950	87557	85674	177260	151550	129530	93060.3	152780	0.609113	DOWN	0.017
Q3UH53	Sdk1	sp|Q3UH53|SDK1_MOUS	1636	1	103.999	2.85E-79	122.66	1	N	TVN(	TVN(100)	3	2	0.21	520	432	119040		109390	170690	223960	175580	114215	190077	0.600889	DOWN	0.042
Q6PGD0	Atraid	sp|Q6PGD0|ARAID_MOU	73	1	82.4373	5.18E-55	115.04	1	N	GTIL(	GTILGLDL	10	2	-2	742	558	193310	216260	243980	428790	360500	312410	217850	367233	0.59322	DOWN	0.015
Q61207	Psap	sp|Q61207|SAP_MOUSE	214	1	141.879	8.7E-121	141.88	1	N	TN(1	TN(140)S	2	2	-1.5	605	478	104440	110810	154180	211740	206340	204970	123143	207683	0.592938	DOWN	0.006
Q8VHY0	Cspg4	sp|Q8VHY0|CSPG4_MOU	2080	1	204.335	1.1E-176	204.34	1	N	LDPT	LDPTVLD	13	2	-0.2	912	690	1061600	973910	982350	1633500	1963800	1507800	1005953	1701700	0.591146	DOWN	0.007
P97927	Lama4	sp|P97927|LAMA4_MOUS	735	1	46.8354	0.009874	85.813	1	N	LFIEE	LFIEEAN(	7	2	2.74	449	386	627150	534370	472930	993630	1016300	755560	544817	921830	0.591016	DOWN	0.016
Q61555	Fbn2	sp|Q61555|FBN2_MOUSE	1749	1	67.6886	2.4E-103	157.54	2	N	SYN(	SYN(80)G	14	2	1.01	635	492	215430	260680	150720	332240	414900	317530	208943	354890	0.588755	DOWN	0.029
Q9WV91	Ptgfrn	sp|Q9WV91|FPRP_MOUS	300	1	139.776	2.84E-93	150.21	1	N	DLDI	DLDLSCN	7	2	2.6	1092	834	1064500	782780	1054200	1616600	1798100	1548000	967160	1654233	0.584658	DOWN	0.004
Q9JKR6	Hyou1	sp|Q9JKR6|HYOU1_MOU	596	1	104.856	1.6E-197	155.28	1	N	EN(1	EN(100)G	2	2	-1.2	1044	796	277670	226790	252290	471360	421760	407180	252250	433433	0.581981	DOWN	0.002
P35330	Icam2	sp|P35330|ICAM2_MOUS	45	0.999922	41.0537	0	158.38	1	N	IN(1)	IN(41)CST	2	2	-0.8	337	258	440890	486490	548930	908940	1052600	636160	492103	865900	0.568314	DOWN	0.041
O54998	Fkbp7	sp|O54998|FKBP7_MOUS	41	1	74.8486	0.004603	96.957	1	N	IEVLI	IEVLHRPE	9	3	-1.1	127	83	150610		178720	320500	291430	267680	164665	293203	0.561607	DOWN	0.01
Q3TEW6	Mpzl1	sp|Q3TEW6|MPZL1_MOU	50	1	105.358	9.03E-25	127.94	1	N	EIFV	EIFVVN(1	6	2	1.69	513	424	226880	345750	335180		606570	472200	302603	539385	0.561015	DOWN	0.043
P97300	Nptn	sp|P97300|NPTN_MOUSE	283	1	183.866	2.85E-92	184.43	1	N	ENG	EN(-180)(	10	2	-1.7	434	375	626850	569130		1086600	1256800	869080	597990	1070827	0.558438	DOWN	0.048
G5E8Q8	Adgrf5	sp|G5E8Q8|AGRF5_MOU	270	1	197.795	0	222.48	1	N	MDY	MDYN(-2	12	2	-0.1	75	46	201150	258430	224670	411010	493910	334410	228083	413110	0.552113	DOWN	0.019
G5E8Q8	Adgrf5	sp|G5E8Q8|AGRF5_MOU	665	1	108.337	0	216.01	1	N	EN(1	EN(110)V	2	2	-1.2	74	46	617330	676730	744860	1184700	1385200	1142400	679640	1237433	0.549234	DOWN	0.003
Q9Z0F8	Adam17	sp|Q9Z0F8|ADA17_MOU	539	1	134.658	2.65E-32	147.78	1	N	CQE	CQEAIN(1	6	2	-4.5	1103	841	157380	209990	220590	334590	454070	282580	195987	357080	0.548859	DOWN	0.041
Q64735	Cr1l	sp|Q64735|CR1L_MOUSE	295	0.999423	32.386	5.22E-12	98.167	1	N	CTPI	CTPPPYV	16	3	0.22	710	533	244740	374890		584490	620160	489520	309815	564723	0.548614	DOWN	0.036
P55065	Pltp	sp|P55065|PLTP_MOUSE	143	1	105.943	1.75E-33	110.44	1	N	VSN(	VSN(110)	3	2	0.55	391	314	344040	339510	343220	664470	664950	557510	342257	628977	0.544148	DOWN	0.001
Q9DBP0	Slc34a2	sp|Q9DBP0|NPT2B_MOU	295	1	54.199	5.68E-82	154.94	1	N	VIQ(	VIQQIAM	14	2	0.44	1007	766	162000	269640	247400	479120	462530	310700	226347	417450	0.542213	DOWN	0.038
Q8K4Q8	Colec12	sp|Q8K4Q8|COL12_MOU	159	0.99891	29.6191	0	200.82	2	N	ETLC	ETLQN(3	5	2	-1.3	866	659	298640	396750	359600	598070	781060	611770	351663	663633	0.529906	DOWN	0.009
Q8K4Q8	Colec12	sp|Q8K4Q8|COL12_MOU	168	1	129.753	0	200.82	2	N	ETLC	ETLQN(3	14	2	-0.9	867	659	298640	396750	359600	598070	781060	611770	351663	663633	0.529906	DOWN	0.009
O08999	Ltbp2	sp|O08999|LTBP2_MOUS	406	1	130.788	6.32E-38	130.79	1	N	DEC	DECWCP	8	2	-0.6	83	56	413810	462420	422290	1040000	826260	621590	432840	829283	0.521945	DOWN	0.031
P55772	Entpd1	sp|P55772|ENTP1_MOUS	73	1	57.2787	6.77E-07	80.815	1	N	EN(1	EN(57)DT	2	2	0.61	398	317	109870	89692	103410	204720		183400	100991	194060	0.520409	DOWN	0.004
Q91ZX7	Lrp1	sp|Q91ZX7|LRP1_MOUSE	4076	1	159.439	4.83E-54	159.44	1	N	LN(1	LN(160)G	2	2	3.32	938	705	866710	969290	876610	1870300	1796600	1696600	904203	1787833	0.505754	DOWN	1E-04
Q5DU41	Lrrc8b	sp|Q5DU41|LRC8B_MOU	78	0.999979	46.6791	7.7E-265	130.78	1	N	GSEI	GSEN(47)	4	2	-1.2	556	450	45891	40548	44520	78843	112480	76347	43653	89223.3	0.489255	DOWN	0.018
Q91ZX7	Lrp1	sp|Q91ZX7|LRP1_MOUSE	3334	1	145.078	1.03E-80	150.44	1	N	TCVS	TCVSN(1	5	2	-3.6	945	705	341790	464360	440280	836820	864380		415477	850600	0.488451	DOWN	0.003
P09055	Itgb1	sp|P09055|ITB1_MOUSE	669	1	238.006	0	278.06	1	N	KDT(	KDTCAQE	12	3	-2.2	214	149	2183700	2324600	2369000	4593400	5608700	3944400	2292433	4715500	0.486149	DOWN	0.008
P11438	Lamp1	sp|P11438|LAMP1_MOUS	177	1	69.6405	1.57E-33	110.44	1	N	DATI	DATIQAY	12	2	-0.6	238	167	300060		326820	680840	670010	584310	313440	645053	0.485913	DOWN	0.004
P28828	Ptprm	sp|P28828|PTPRM_MOUS	534	1	59.731	1.44E-21	100.07	1	N	AVSS	AVSSFDP	13	2	-1.4	320	247	95573		142330	233150	293850	216670	118952	247890	0.479856	DOWN	0.035
P37889	Fbln2	sp|P37889|FBLN2_MOUS	497	1	90.3757	9.16E-87	120.75	1	N	EGET	EGETCGA	10	2	-1.7	348	266	42333	56036	47003	114880	105900	83588	48457.3	101456	0.477619	DOWN	0.006
Q61469	Plpp1	sp|Q61469|PLPP1_MOUS	142	1	161.037	2.5E-282	168.69	1	N	IN(1)	IN(160)CS	2	2	0.72	617	485	183430	298330	296650	584980	633640	436840	259470	551820	0.470208	DOWN	0.014
P56528	Cd38	sp|P56528|CD38_MOUSE	223	1	193.464	0	193.62	1	N	N(1)S	N(190)STI	1	2	1.89	401	320	465800	476220	576370	1056300	1357600	815430	506130	1076443	0.470187	DOWN	0.024
D3YXG0	Hmcn1	sp|D3YXG0|HMCN1_MOU	3220	1	74.5013	0	256.4	1	N	SNS(	SN(-75)S	5	2	-1.6	58	36	120870	180200	148030	404260	314610	245270	149700	321380	0.465804	DOWN	0.025
O88207	Col5a1	sp|O88207|CO5A1_MOU	1672	1	93.6492	4.6E-148	164.22	1	N	VYCI	VYCN(94)	4	2	2.78	143	97	100690	83436	94648	199970	183250	217840	92924.7	200353	0.463804	DOWN	7E-04
P09055	Itgb1	sp|P09055|ITB1_MOUSE	212	1	175.328	0	285.99	1	N	NPC	N(-180)P	8	2	0.59	217	149	1922500	3438000	2828700	7128800	6757600	4154700	2729733	6013700	0.453919	DOWN	0.034
Q9R0L9	Cd164	sp|Q9R0L9|MUC24_MOU	103	1	169.98	0	199.3	1;2	N	TYCA	TYCAN(-	15	2	-0.1	1069	817	410500	404550	452600	975880	1052700	768780	422550	932453	0.453159	DOWN	0.004
P97370	Atp1b3	sp|P97370|AT1B3_MOUS	197	1	129.045	1.7E-198	159.82	1	N	EEN(	EEN(130)	3	2	0.74	440	379	700760	639260	642330	1266300	1685100	1430100	660783	1460500	0.452436	DOWN	0.003
Q9R0L9	Cd164	sp|Q9R0L9|MUC24_MOU	98	1	126.102	0	199.3	2	N	TYCA	TYCAN(-	10	2	-1.1	1068	817	407920	404550	452600	975880	1052700	768780	421690	932453	0.452237	DOWN	0.004
P09470	Ace	sp|P09470|ACE_MOUSE	700	1	90.2625	1.28E-16	135.63	1	N	STEV	STEVSN(	6	2	-6.6	222	152	2325200	2099300	2702300	4728400	6831500	4208300	2375600	5256067	0.451973	DOWN	0.025
P15208	Insr	sp|P15208|INSR_MOUSE	242	1	93.1623	4.2E-70	117.21	1	N	ECLC	ECLGN(9	5	2	-2.2	258	185	28822	37243		73735	86826	58955	33032.5	73172	0.451436	DOWN	0.034
Q91ZX7	Lrp1	sp|Q91ZX7|LRP1_MOUSE	3663	0.958689	13.6561	3.7E-104	147.02	1	N	TCPL	TCPLDEF	10	2	-1.3	944	705	264100	196930		501650	525270		230515	513460	0.448944	DOWN	0.015
Q6GU68	Islr	sp|Q6GU68|ISLR_MOUSE	309	1	106.466	1.76E-60	137.39	1	N	FQA	FQAFAN(	6	2	2.3	728	545	237100	129430	152260	520020	291770	364760	172930	392183	0.440942	DOWN	0.043
Q8BYW9	Eogt	sp|Q8BYW9|EOGT_MOUS	354	1	67.4639	0.000385	110.22	1	N	LN(1	LN(67)ITC	2	2	-1.7	829	624		177950	155780	456860	345560	339570	166865	380663	0.438353	DOWN	0.023
P20352	F3	sp|P20352|TF_MOUSE Tis	37	1	99.3405	1.69E-33	116.13	1	N	AFN	AFN(99)L	3	2	0.32	290	219	100640	159250		311290	302950	294900	129945	303047	0.428795	DOWN	0.005
O09118	Ntn1	sp|O09118|NET1_MOUSE	95	1	65.9326	0.000122	77.358	1	N	SCHI	SCHLCN(	6	2	-8	91	60	37453	45267	33567	97119	101410	73826	38762.3	90785	0.426968	DOWN	0.005
P28828	Ptprm	sp|P28828|PTPRM_MOUS	249	1	68.3716	9.9E-43	132.44	1	N	FIASI	FIASFN(-	9	2	2.09	322	247	83478	87849	88603	249820	157120	203560	86643.3	203500	0.425766	DOWN	0.012
Q08857	Cd36	sp|Q08857|CD36_MOUSE	321	0.990035	19.9719	0	274.44	1	N	VISN	VISN(-20)	5	2	2.6	505	419	5769800	5724200	6496800	1.3E+07	1.6E+07	1.3E+07	5996933	1.4E+07	0.421557	DOWN	0.002
B2RU80	Ptprb	sp|B2RU80|PTPRB_MOUS	199	1	119.79	4.49E-77	119.79	1	N	SFPV	SFPVYIN(	7	2	-2.1	28	16	212640		252580	557390	587090	526850	232610	557110	0.41753	DOWN	0.001
P16406	Enpep	sp|P16406|AMPE_MOUSE	601	1	85.8837	0	230.23	1	N	GGIT	GGITLN(-	8	2	-2.2	270	196	1232900	792760	1015000	2751800	2044300	2538700	1013553	2444933	0.414553	DOWN	0.004
Q3V3R4	Itga1	sp|Q3V3R4|ITA1_MOUSE	839	1	45.4892	4.8E-221	199.51	1	N	FN(1	FN(45)VS	2	2	-0.4	541	444	273150		377640	925450	862350	624830	325395	804210	0.404614	DOWN	0.031
Q6F3F9	Adgrg6	sp|Q6F3F9|AGRG6_MOU	477	0.80302	6.10303	6.8E-120	157.77	1	N	LMTI	LMTN(6.1	4	2	-2.1	722	542		247520	259840	628700		629920	253680	629310	0.403108	DOWN	3E-04
Q64487	Ptprd	sp|Q64487|PTPRD_MOUS	724	1	47.3342	2.7E-119	149.02	1	N	VEVE	VEVEAVN	7	2	-3.9	708	530	82689	93427	117060	254220	262230	214560	97725.3	243670	0.401056	DOWN	0.001
P97333	Nrp1	sp|P97333|NRP1_MOUSE	261	1	76.0093	1.1E-115	108.34	1	N	EGFS	EGFSAN(	6	2	0.55	437	378		241730	292140	621710	713260		266935	667485	0.399912	DOWN	0.017
Q8VIB3	St3gal6	sp|Q8VIB3|SIA10_MOUSE	308	0.996441	24.4712	1.99E-87	153.18	1	N	QN((	QN(-24)A	6	3	-2.7	918	692	59972	52091	56946	183570	132310	109240	56336.3	141707	0.397556	DOWN	0.018
P55284	Cdh5	sp|P55284|CADH5_MOUS	523	1	99.0052	5.4E-302	207.41	2	N	NED	N(-99)ED	5	3	-1.1	392	315	168810		226810	489240	616590	442290	197810	516040	0.383323	DOWN	0.02
D3YXG0	Hmcn1	sp|D3YXG0|HMCN1_MOU	2886	1	172.087	1.9E-203	172.09	1	N	GAN	GAN(-17	15	2	-3.4	54	36	566330	775560	785110	1999900	1832300	1748800	709000	1860333	0.381114	DOWN	4E-04
Q3V3R4	Itga1	sp|Q3V3R4|ITA1_MOUSE	1113	1	164.59	0	233.59	1	N	GELC	GELQSEN	7	2	-0.3	542	444	1139900	1337100	1167000	3371700	3638500	2568900	1214667	3193033	0.380412	DOWN	0.004
Q08481	Pecam1	sp|Q08481|PECA1_MOUS	540	1	129.314	3.9E-131	164.56	1	N	EDRI	EDRPFHQ	11	4	-0.8	499	418	113950	125260	87599	361320	308750	199630	108936	289900	0.375772	DOWN	0.021
Q8R5M8	Cadm1	sp|Q8R5M8|CADM1_MO	104	1	142.709	1.5E-293	164.31	1	N	FQLI	FQLLN(14	5	2	1.35	889	673	1550800	1812800	1936400	4530200	5640200	4176700	1766667	4782367	0.369413	DOWN	0.003
Q00651	Itga4	sp|Q00651|ITA4_MOUSE	487	1	103.634	1.5E-115	130.12	1	N	TRP\	TRPVVIVE	18	3	0.43	459	394	83074		58580	206030	166120	214930	70827	195693	0.361929	DOWN	0.01
P97333	Nrp1	sp|P97333|NRP1_MOUSE	150	1	86.7287	1.84E-81	153.18	1	N	RGPI	RGPECSQ	8	3	2.41	438	378	530010	449350	469350	1365100	1480800	1191700	482903	1345867	0.358805	DOWN	6E-04
Q62469	Itga2	sp|Q62469|ITA2_MOUSE	102	1	157.296	0	254.47	1	N	LNL(	LN(-220)L	11	2	1.23	680	519	221370	263100	254640	829770	798580	444060	246370	690803	0.356643	DOWN	0.023
Q3V3R4	Itga1	sp|Q3V3R4|ITA1_MOUSE	217	0.999989	49.9158	0	312.39	1	N	QTQ	QTQVGIV	12	3	-2.7	546	444	1138400	1398500	1321700	4607600	3939000	2695600	1286200	3747400	0.343225	DOWN	0.012
P47801	Emp1	sp|P47801|EMP1_MOUSE	43	0.999998	58.0121	0	193.26	1	N	N(1)(	N(58)CTG	1	2	-1	362	279	206100	166730	152380	598950	549250	386110	175070	511437	0.34231	DOWN	0.007
P09470	Ace	sp|P09470|ACE_MOUSE	719	1	81.8652	0	173.07	1	N	TFD\	TFDVSN(-	9	2	1.11	223	152	668450	857380	853130	2344700	2920400	1853900	792987	2373000	0.334171	DOWN	0.007
Q9DAW9	Cnn3	sp|Q9DAW9|CNN3_MOU	240	1	122.936	0	186.7	1	N	LTLC	LTLQPVD	8	2	1.24	1004	762	71887	94940	75031	352570	215740	183960	80619.3	250757	0.321504	DOWN	0.031
Q3V3R4	Itga1	sp|Q3V3R4|ITA1_MOUSE	100	1	167.012	1.9E-249	169.52	1;2	N	LDLF	LDLPVN(1	6	2	0.98	544	444	3485200	2521300	2862600	8157400	1.1E+07	9153100	2956367	9509833	0.310875	DOWN	0.002
P15209	Ntrk2	sp|P15209|NTRK2_MOUS	254	1	56.2022	3E-15	85.622	1	N	ITN(	ITN(56)IS	3	2	1.81	261	186	62778		43770	150350	227500	138810	53274	172220	0.309337	DOWN	0.048
P28843	Dpp4	sp|P28843|DPP4_MOUSE	514	1	103.804	9.8E-228	223.96	1	N	LDFI	LDFIVLN(	7	2	-3.9	326	248	15741	30880	46737	127760	110580	63548	31119.3	100629	0.309247	DOWN	0.03
P09470	Ace	sp|P09470|ACE_MOUSE	514	1	87.895	8.36E-08	114.39	1	N	N(1)I	N(88)ETH	1	2	-3.8	221	152	309820	326790	349970	1064500	1250400	993920	328860	1102940	0.298167	DOWN	6E-04
Q08857	Cd36	sp|Q08857|CD36_MOUSE	417	1	56.4735	0	227.73	1	N	RPYI	RPYIVPIL	11	2	1.25	503	419	1265900	1585700	2292900	7290200	4042800	5929200	1714833	5754067	0.298021	DOWN	0.015
Q3V3R4	Itga1	sp|Q3V3R4|ITA1_MOUSE	105	1	167.012	1.1E-105	167.01	1;2	N	LDLF	LDLPVN(1	11	2	0.98	545	444	689450	547810	566530	1649700	2450200	1994700	601263	2031533	0.295965	DOWN	0.004
Q61739	Itga6	sp|Q61739|ITA6_MOUSE	731	0.997924	26.8585	3.5E-113	115.24	1	N	QLS(	QLSCVAN	9	2	-0.9	656	501	65018	90466	53762	289090	286120	149730	69748.7	241647	0.288639	DOWN	0.022
Q8R5M8	Cadm1	sp|Q8R5M8|CADM1_MO	70	1	199.298	0	199.3	1	N	DVT\	DVTVIEGI	17	2	-1.5	888	673	332340	389230	421330	1000800	2040000	1087700	380967	1376167	0.276832	DOWN	0.041
Q3V3R4	Itga1	sp|Q3V3R4|ITA1_MOUSE	418	1	141.28	0	216.01	1	N	ANQ	AN(-140)	9	2	-0.1	538	444	771070	889360	665210	3504200	3236900	1765200	775213	2835433	0.273402	DOWN	0.019
Q8VHR0	Pcdh18	sp|Q8VHR0|PCD18_MOU	420	1	114.401	4.7E-88	132.93	1	N	TYEN	TYEN(-12	11	2	-4.2	911	689		67695	61614	246600	262440	220170	64654.5	243070	0.265991	DOWN	0.002
Q3V3R4	Itga1	sp|Q3V3R4|ITA1_MOUSE	459	1	76.572	2.68E-48	159.93	1	N	YN(1	YN(77)HT	2	2	-2	548	444	132650	207940	119400	977250	706320	606600	153330	763390	0.200854	DOWN	0.006
Q9R1W5	Calcrl	sp|Q9R1W5|CALRL_MOU	65	1	73.502	0	257.38	1	N	IMQ	IMQDPIQ	15	2	-1.5	1071	821	970570	1199300	1448900	7015400	6650500	4819800	1206257	6161900	0.195761	DOWN	0.002
Q5FWI3	Cemip2	sp|Q5FWI3|CEIP2_MOUS	525	1	110.906	1.3E-104	142.08	1	N	N(1)	N(110)FT	1	2	-0.7	560	451	21448	26900	19290	114880	132210	108050	22546	118380	0.190454	DOWN	2E-04
Q9R1W5	Calcrl	sp|Q9R1W5|CALRL_MOU	128	0.956455	13.4173	1.4E-116	141.88	2	N	TWT	TWTN(92	10	2	-1.6	1074	821	50564	39202	51421	331240	389840	370680	47062.3	363920	0.129321	DOWN	6E-05
Q9R1W5	Calcrl	sp|Q9R1W5|CALRL_MOU	122	1	91.7625	1.4E-116	141.88	1;2	N	TWT	TWTN(92	10	2	-1.6	1072	821	50564	39202	51421	331240	428900	370680	47062.3	376940	0.124854	DOWN	3E-04

S2 Table. The venn diagram analysis of differentially expressed proteins.

Protein-UP	Protein-DOWN	N-glycosylated proteins-UP	N-glycosylated proteins-DOWN	P-UP|N-UP	P-DOWN|N-DOWN
Akr1b8	Selenon	Cma1	Stt3b	Cdh13	Ace
Ckmt1	Mtss2	Hp	Plxnb2	Col6a2	Cd36
Ephx1	Ndufb10	Itgae	Itga3		Itga2
Phlda3	Cggbp1	Osmr	Lrp1		Itga1
Dglucy	Usp19	Tlr13	Calu		Cadm1
Scd1; Scd3; S	Aqp5	Serpina3m	Cacna2d1		Enpep
Anxa8	Gtf2b	Cfh	Hyou1		
Reg3g	Cdk14	Cdh13	Fat4		
Aacs	Sparcl1	Fcgr2	Cacna2d1		
Mb	Ndufb8	Sulf1	Tmem87a		
Vill	Fmo1	Msln	Tor1b		
Itgb4	Ace	Lgals3bp	Lrp1		
Cd109	Tst	Adamtsl4	Aoc3		
Ak1	Cbx3	Col6a2	Plxnc1		
Postn	Pgm5	Cfh	Robo4		
Slc25a35	Gfra2	C5	Il6st		
Aldh1a2	Abca3		Plxnb1		
Aldh1a1	Macroh2a2		Lrp4		
Col12a1	Snrnp70		Ptdss2		
Mif	Lyn		Plxnb2		
Mmp2	Mtarc2		Sdk1		
Itih5	Gucy1b1		Atraid		
Arhgap35	Itgb2		Psap		
Sod2	Lbr		Cspg4		
Cnn1	Hmgb2		Lama4		
Lum	H4c1		Fbn2		
Dcxr	Tek		Ptgfrn		
Ugt1a6	Adgre5		Hyou1		
Col18a1	Ndufb11		Icam2		
Ldhb	Sdhb		Fkbp7		
Tgfbr2	Cd36		Mpzl1		
Oat	Itga2		Nptn		
Bgn	Ndufb7		Adgrf5		
Fdps	Thbd		Adgrf5		
Gsto1	Stom		Adam17		
Cdh13	Mme		Cr1l		
Sfxn1	Mpp2		Pltp		
Cox5a	Mtr		Slc34a2		
Cyp2s1	Tppp		Colec12		
Pccb	Pag1		Colec12		
Wfdc2	Ank2		Ltbp2		
Uap1l1	Cd74		Entpd1		
Gcdh	Cmpk2		Lrp1		
Epha2	Basp1		Lrrc8b		
Mmrn2	Naalad2		Lrp1		
Impact	Ndufb5		Itgb1		
Nes	Alkbh5		Lamp1		
Ass1	Itga1		Ptprm		
Sfn	Cadm1		Fbln2		
Rabggta	Sema7a		Plpp1		
Adck1	Enpep		Cd38		
Col4a2	Ndufa10		Hmcn1		
Kif1c	Ndufa3		Col5a1		
Dpt	Stfa2		Itgb1		
Col6a1	Pdgfrb		Cd164		
Adss1	Ndufa5		Atp1b3		
Rab34	Ndufa13		Cd164		
Inf2	Psmb9; Psmb9		Ace		
Aldh1a7	H1-2		Insr		
Mat1a	Ndufa11		Lrp1		
Cyp2f2	Ndufs7		Islr		
Dysf	Ndufs3		Eogt		
Acss2	B2m		F3		
Efemp1	Elp2		Ntn1		
Slc25a3	Reck		Ptprm		
Col15a1	Ndufa9		Cd36		
Gclc	Ndufs2		Ptprb		
Wdr35	Ndufs5		Enpep		
Scrn1	Dchs1		Itga1		
Igf2r	H1-3		Adgrg6		
Col6a2	Tubb1		Ptprd		
Stab1	H1-5		Nrp1		
	H1-1		St3gal6		
	Bin2		Cdh5		
	Gpihbp1		Hmcn1		
	Gap43		Itga1		
	Ndufs6		Pecam1		
	Lyz1		Cadm1		
	H1-4; H1-6		Itga4		
	Kit		Nrp1		
	Ndufs4		Itga2		
	Ndufv1		Itga1		
	Aco1		Emp1		
	Ndufs1		Ace		
	Ndufv2		Cnn3		
	Vsnl1		Itga1		
	Ndufa2		Ntrk2		
	Ndufa12		Dpp4		
			Ace		
			Cd36		
			Itga1		
			Itga6		
			Cadm1		
			Itga1		
			Pcdh18		
			Itga1		
			Calcrl		
			Cemip2		
			Calcrl		
			Calcrl		
