# Supplementary material for: N-glycoproteomic profiling reveals aberrant ITGB1 glycosylation in hyperoxia-induced bronchopulmonary dysplasia
Source: PLoS One. 2026 Nov 10;21(11):e0362192. doi: 10.1371/journal.pone.0362192 (PMC13750597; doi:10.1371/journal.pone.0362192)

uncropped membrane

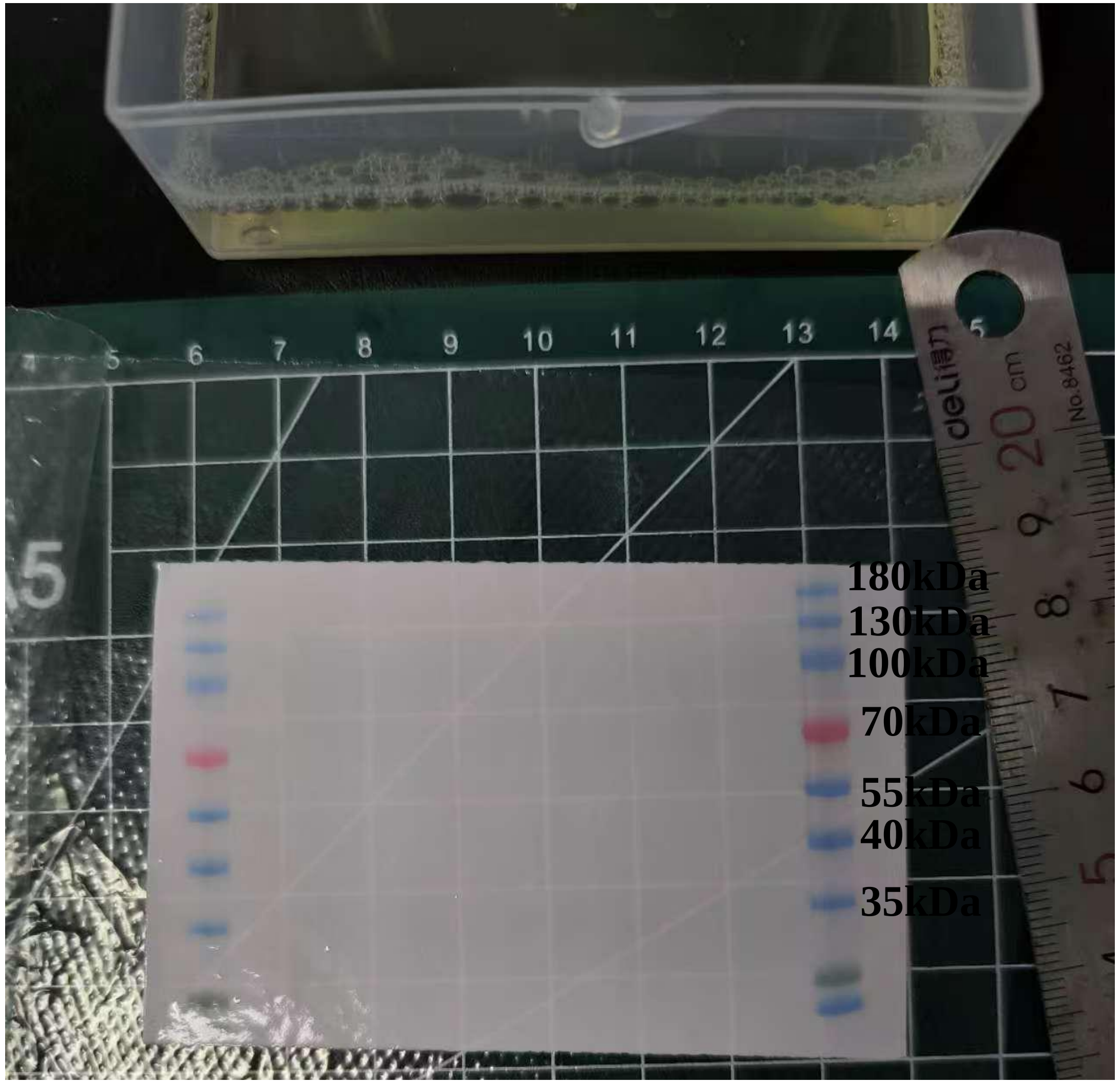

cropped membrane

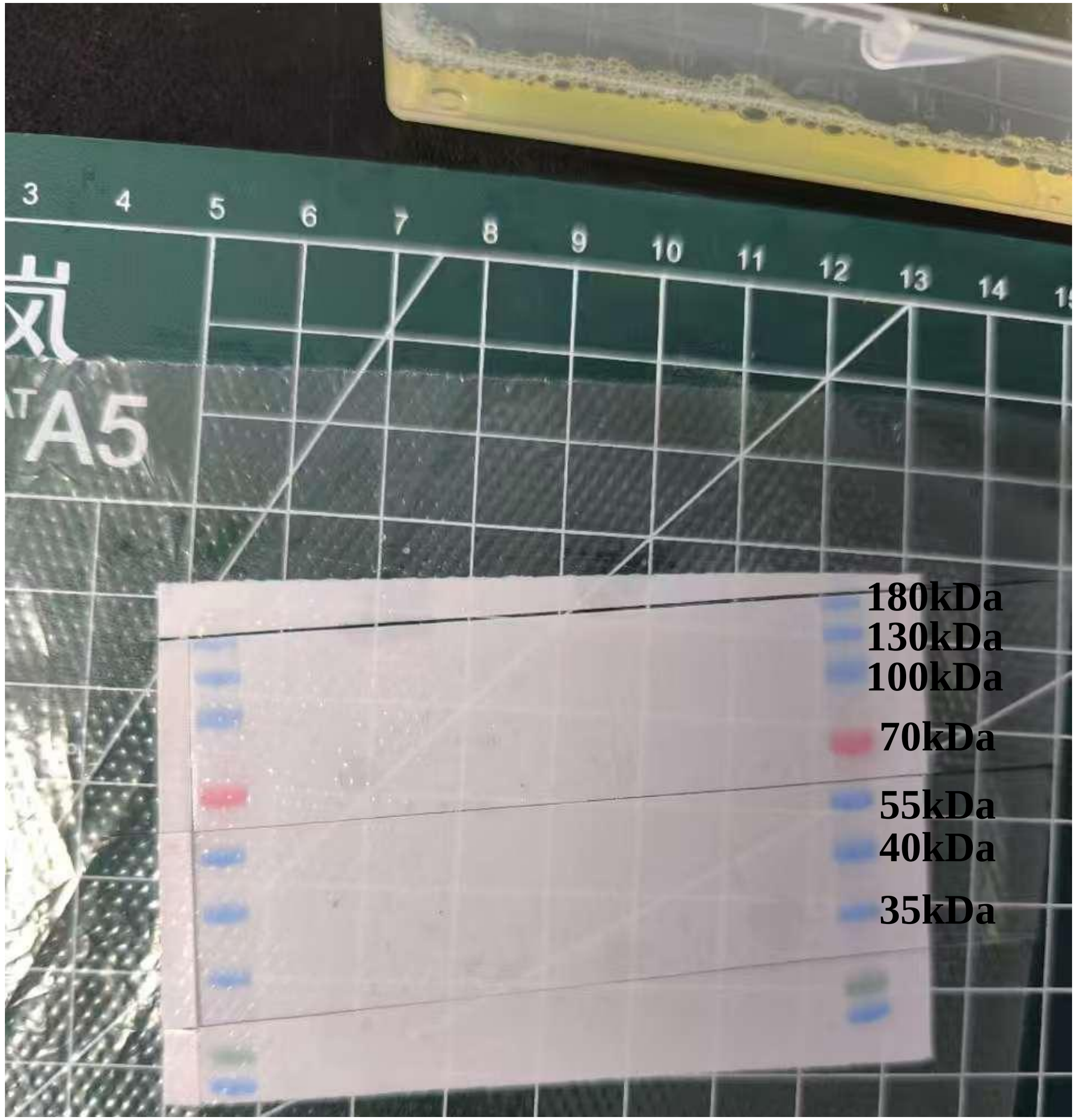

cropped images used in the main figure

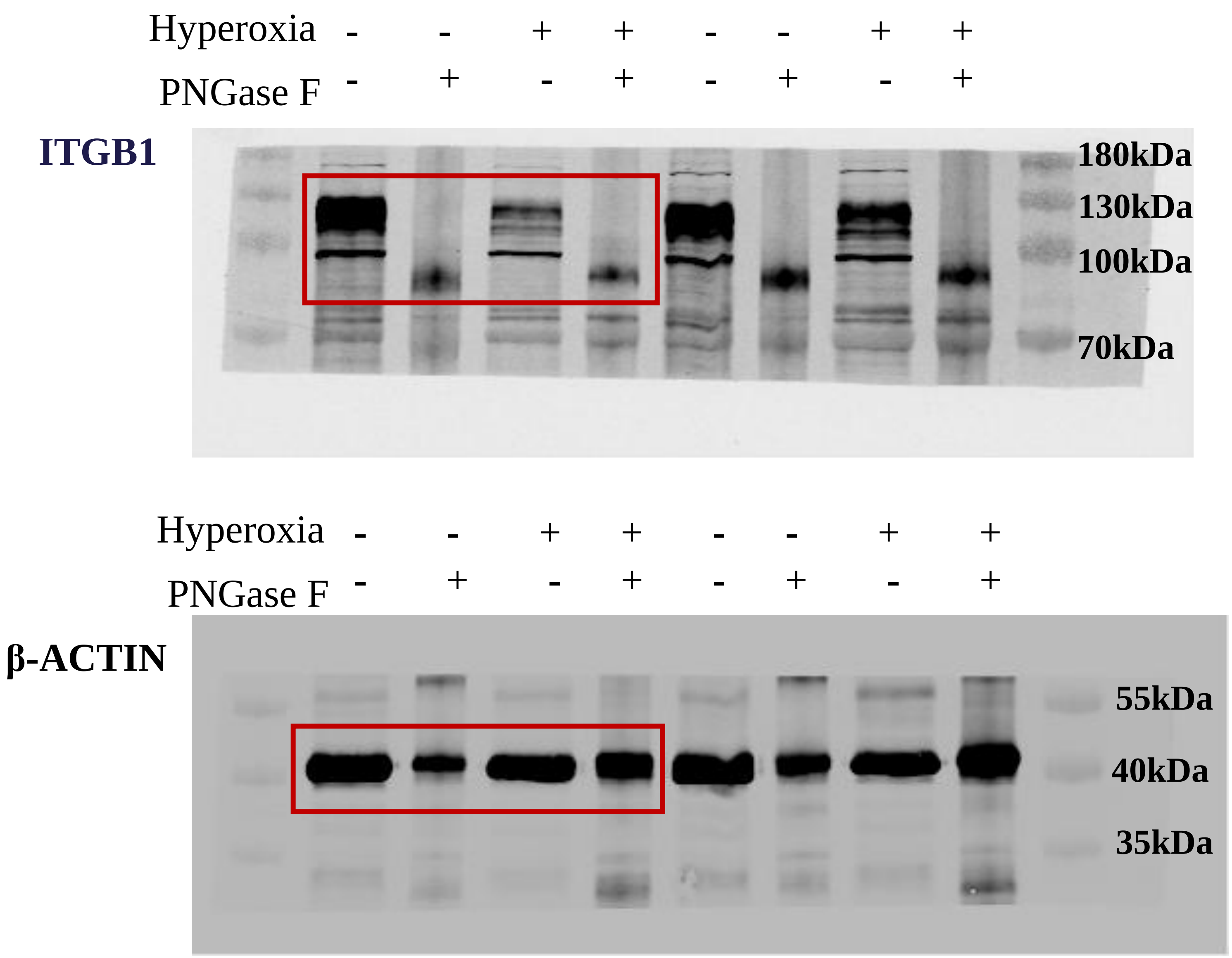

Supplement: Supplementary file 2 [file pone.0362192.s002.pdf]
